# Dissecting Molecular Interactions in Aqueous Deep Eutectic Solvents: A Multi-Scale Study of Choline- and Acetylcholine-Based Propylene Glycol Isomers

**DOI:** 10.1021/acs.jpcb.5c08377

**Published:** 2026-02-16

**Authors:** Dorota Warmińska, Adrianna Sutkowska, Marzena Jamrógiewicz, Piotr Cysewski

**Affiliations:** † Department of Physical Chemistry, Faculty of Chemistry, 49557 Gdańsk University of Technology, ul. Narutowicza 11/12, 80-233 Gdańsk, Poland; ‡ Department of Physical Chemistry, Faculty of Pharmacy, Medical University of Gdańsk, Al. Gen. Hallera 107, 80-416 Gdańsk, POLAND; § Department of Physical Chemistry, Pharmacy Faculty, Collegium Medicum of Bydgoszcz, 431465 Nicolaus Copernicus University in Toruń, Kurpińskiego 5, 85-096 Bydgoszcz, Poland

## Abstract

Deep eutectic solvents (DESs) based on choline or acetylcholine chlorides with 1,2- or 1,3-propanediol represent promising green alternatives to conventional solvents, yet their thermodynamic behavior is governed by a complex interplay of intermolecular forces. To clarify how water modulates their molecular organization and thermodynamic behavior, this study combines experimental, theoretical, and computational approaches. High-precision density and sound velocity are presented for four DESs—composed of choline chloride (ChCl) or acetylcholine chloride (AChCl) with 1,2-propanediol (1,2-PG) or 1,3-propanediol (1,3-PG)—mixed with water across the entire composition range from 293.15 to 313.15 K. From these data, excess molar volumes and excess isentropic compressibilities were obtained and correlated using Redlich–Kister equations. In addition, Prigogine–Flory–Patterson (PFP) analysis revealed that negative excess functions originate predominantly from strong hydrogen-bond interactions, whose magnitude follows the order AChCl:1,2-PG < ChCl:1,2-PG < AChCl:1,3-PG < ChCl:1,3-PG. Consequently, the interactional contribution, rather than free volume effects, dominates excess molar volume. At the molecular level, a COSMO-RS-based speciation model was developed, quantifying the shifting equilibrium of homo- and heteromolecular pairs and directly linking the stronger self-association of 1,3-PG and AChCl to their attenuated volumetric contraction upon mixing with water. Furthermore, a robust linear regression model was constructed using COSMO-RS molecular descriptors, enabling the accurate prediction of aqueous DES density and revealing the dominant role of glycol self-association and hydration energetics. Together, these results provide a consistent multiscale picture from macroscopic properties to molecular distributions, offering a predictive pathway for the rational design of aqueous DES systems.

## Graphical abstract

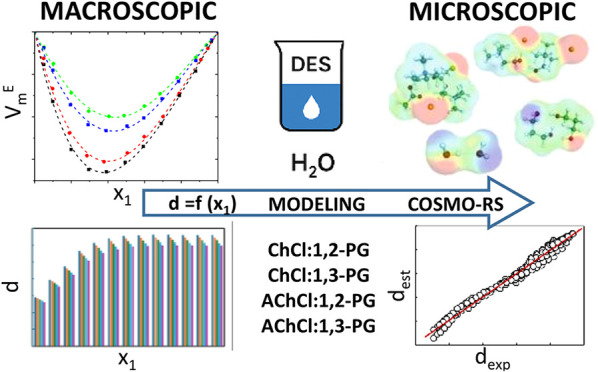

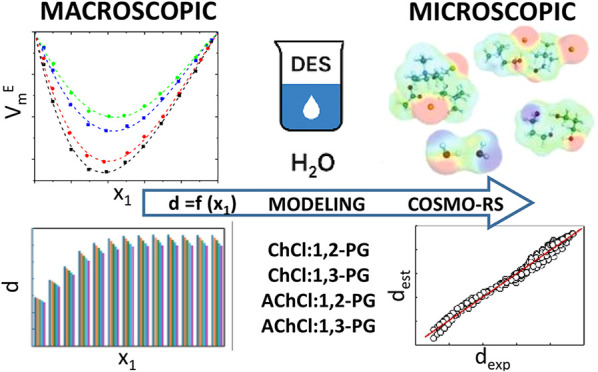

## 1 Introduction

Deep eutectic solvents (DESs) have emerged over the past two decades as a novel class of green solvents characterized by ease of synthesis, low toxicity, biodegradability, and tunable physicochemical properties.^1–3^ Typically formed by mixing a hydrogen bond acceptor (HBA) with a hydrogen bond donor (HBD), DESs exhibit a significant depression in their melting point compared to that of their components, resulting in a stable liquid phase at or near room temperature. Since their introduction by Abbott et al., DESs—particularly those based on choline chloride (ChCl) due to its low cost, nontoxic, and biodegradable character—have garnered considerable attention for applications in catalysis, extraction, electrochemistry, and pharmaceutical formulations, to name a few.^4–6^ However, the higher viscosity of many DESs than most common molecular solvents limits their practical use, especially in processes requiring efficient mass and heat transfer. To overcome this, DESs are often mixed with molecular solvents such as water, alcohol, or acetonitrile to adjust their properties.

Among the others, aqueous mixtures of DESs are of growing interest due to their relevance in biological, pharmaceutical, and environmental fields. Water, being the most abundant, low-viscous, and environmentally friendly solvent, not only affects the physicochemical properties of DESs but also influences their hydrogen bonding network and solvation dynamics, thereby expanding their application scope. For instance, Chourasia et al. found that the addition of water as a cosolvent enhances the effectiveness of DES in delignification.^7^ Lien et al. discovered that the presence of water in acetamide:lithium perchlorate improves the flame-retardant property of DES.^8^ Posada et al. demonstrated that the addition of water to DES enhances the conditions for synthesizing nanoporous materials.^9^ Investigating the thermodynamic behavior of DES-water systems is crucial for the rational design of solvent systems tailored for specific applications, including drug delivery, extraction processes, and reaction media.^10–12^

A detailed analysis of physicochemical properties, supported by theoretical and computational studies, provides valuable insights into the molecular interactions and structural organization within DES-based mixtures. In recent years, several research groups have published reports on the physical properties of aqueous solutions of ChCl-based DESs, the most popular mixtures used for pharmaceutical purposes.^13^ Most of them have published reports on the density and viscosity of mixtures containing urea, glycerol, and ethylene glycol as HBD.^14–17^ Fewer studies have been conducted on aqueous solutions of DES consisting of ChCl and carboxylic acid or polyols. These include mainly reports on the density of aqueous solutions of ChCl:malonic acid, ChCl:glutaric acid, ChCl:lactic acid, ChCl:oxalic acid, ChCl:acetic acid, ChCl: triethylene glycol, ChCl:butanediol.^18–22^ For all systems studied so far, negative excess molar volumes have been observed, indicating that the hydrogen bonds between water and DES are slightly stronger than those between pure water and pure DES. From experimental studies performed by Hammond et al. and Dai et al., based on neutral total scattering and empirical potential structure refinement, as well as NMR, FTIR, and Raman spectroscopy, and theoretical research based on molecular dynamics simulations, it is known that water forms nanostructures with HBA-H2O-HBD motifs. However, the excessive water addition beyond 40 wt % causes this nanostructure to collapse, resulting in the complete hydration of both HBA and HBD.^23–25^ Thus, the aqueous solutions of DES should be more accurately described as “pseudo-binary mixtures,” as they are treated as binary mixtures, although they are indeed three-component systems.

Among the polyol-based DESs, ChCl:propylene glycol has gained special attention due to its application in health-related areas such as pharmaceutics, foods, and cosmetics.^26,27^ This is because, compared to other glycols, such as ethylene glycol or polyethylene glycols, it is less toxic and less viscous. However, regarding the physicochemical properties of aqueous mixtures of DESs based on 1,2-propanediol (1,2-PG), only two studies have been published, one of which was characterized by high measurement uncertainty.^28,29^ To our knowledge, aqueous solutions of DESs containing acetylcholine chloride (AChCl) as HBA have not yet been reported. Acetylcholine chloride can be considered a structural derivative of choline chloride in which the hydroxyl group is replaced by an acetyl group, forming an ester functional group. This substitution significantly alters its physicochemical properties. Unlike ChCl, which, due to its hydroxyl group, acts as a strong hydrogen bond donor and acceptor, AChCl essentially loses its donor capacity and acts primarily as a hydrogen bond acceptor - through both the chloride anion and the carbonyl oxygen of the ester group. These structural differences are expected to influence the hydrogen bond network and, consequently, the interaction of AChCl-based DESs with water. Understanding these interactions is one of the key goals of this study.

Thus, in this work, four deep eutectic solvents (DESs) were prepared using ChCl and AChCl as HBAs, and 1,2-PG and 1,3-PG as HBD, at a 1:3 molar ratio of HBA to HBD. The densities and speeds of sound of both pure DESs and mixtures with water were measured across the whole concentration range at temperatures from 293.15 to 313.15 K. Moreover, a linear regression model based on five molecular descriptors derived using the Conductor-like Screening Model for Real Solvents (COSMO-RS) was applied to estimate solution densities, which has not been attempted previously. Thermodynamic excess properties, including excess molar volume and excess isentropic compressibility, were calculated and correlated using the Redlich–Kister and Prigogine–Flory–Patterson equations. The effect of HBA/HBD type and temperature on these properties was investigated. The experimental results were further interpreted in terms of molecular interactions between the solution components, supported by COSMO-RS-based theoretical predictions, which shed light on the varying extent of interactions between components in binary mixtures.

## 2 Experimental Section

### 2.1 Chemicals

The chemicals used in this study -1,2-propanediol, 1,3-propanediol, and choline chloride-were purchased from Sigma-Aldrich, while acetylcholine chloride was procured from Acros Organics. The salts were dried under reduced pressure for several days before use, at 323.15 K for ChCl and 298.15 K for AChCl. HBDs were used as received. The corresponding information and the chemical structures of the DES components are presented in Table 1.

**Table 1:** Specification of Chemicals Used In This Work

chemical name	*M*_DES_/(g·mol^–1^)	CAS	supplier	mass fraction purity^a^
choline chloride	139.62	67-48-1	Sigma-Aldrich	>0.98
acethylcholine chloride	181.66	60-31-1	Acros Organics	0.99
1,2-propanediol	76.09	57-55-6	Sigma-Aldrich	>0.995
1,3-propanediol	76.09	504-63-2	Sigma-Aldrich	0.98

a As stated by supplier.

### 2.2 Preparation of DESs and Their Aqueous Solutions

DESs with the molar ratio of 1:3 salt to glycol were prepared by mass. Weighing was performed using an analytical balance (Mettler Toledo) with a precision of 0.1 mg. The standard uncertainty in the mass fraction was estimated to be less than ± 1 × 10^–4^. Quaternary ammonium salts and glycols were mixed at 343.15 K for 1 h using a magnetic stirrer in a fume hood until a homogeneous liquid without any precipitate was obtained. The water content of DESs was measured using a Mettler Toledo Volumetric Karl Fischer titrator (V10S). Table 2 presents its values along with the molar mass of DESs, their abbreviations, and the molar ratio and molar fraction of DES components.

**Table 2:** Summary of Properties of Prepared DESs

abbervation	HBA	HBD	HBA:HBD molar ratio^a^	water content *w*(H_2_O)^b^	*M*_DES_/(g·mol^–1^)
ChCl:1,2-PG	choline chloride	1,2-propanediol	1:3.000	0.00050	91.97
ChCl:1,3-PG	choline chloride	1,3-propanediol	1:2.997	0.00068	91.99
AChCl:1,2-PG	acethylcholine chloride	1,2-propanediol	1:3.018	0.00050	102.36
AChCl:1,3-PG	acethylcholine chloride	1,3-propanediol	1:3.026	0.00100	102.31

a The standard uncertainty of DES mass fraction composition is 0.0001.

b Water content of DESs in mass fraction determined by Karl Fisher titration with the standard uncertainty ± 0.0001.

For the preparation of aqueous mixtures of DESs, deionized, double-distilled, degassed water with a specific conductance of 1.15 × 10^–6^ S·cm^–1^ was used. The water content in the DES was taken into account during the preparation of the solution.

### 2.3 Density and Speed of Sound Measurement

Measurements of density and speed of sound were performed using a digital vibration-tube analyzer (Anton Paar DSA 5000, Austria) with proportional temperature control, which maintained the samples at working temperature with an accuracy of 0.01 K. The experimental frequency for the ultrasonic speed measurements was 3 MHz. The apparatus was calibrated using double-distilled, deionized, and degassed water, as well as dry air at atmospheric pressure, as specified in the apparatus catalog procedure. The uncertainties of density and ultrasonic velocity measurements were better than 0.1 kg·m^–3^ and 0.5 m·s^–1^, respectively.

### 2.4 Isobaric Heat Capacity Measurement

The isobaric heat capacity of DESs was measured using a Mettler Toledo Star One Differential Scanning Calorimeter (DSC), calibrated with the indium standard. Measurements were performed under an inert nitrogen atmosphere with a flow rate of 60 mL·min^–1^ using the sapphire method.^30^ A “baseline” or blank measurement was carried out at a heating rate 10 K·min^–1^. All recorded results were blank curve-corrected, and each measurement was performed in duplicate to ensure reproducibility. The samples, as well as the reference material, were placed in individual aluminum crucibles, which were sealed with pierced lids. The DSC data were recorded and then analyzed to determine the isobaric heat capacity.

## 3 Theoretical and Computational Analysis

### 3.1 Thermodynamic Background

Thermodynamic excess functions are quantities that indicate the deviation of real solutions from ideal behavior and are commonly used to analyze the nature and type of interactions occurring within the system.

The excess molar volume (*V*_*m*_^*E*^) is calculated directly from density measurement according to eq 1:

1 $$V_{m}^{E}=V_{m}-V_{m}^{\mathrm{id}}=\sum_{i}x_{i}M_{i}\left(\frac{1}{d}-\frac{1}{d_{i}}\right)$$

where *d* is the density of the mixture and *x*_*i*_, *M*_*i*_, and *d*_*i*_ are the mole fraction, the molar mass, and the density of pure component *i*, respectively.

The excess molar isentropic compressibility (*K*_*S*,*m*_^*E*^) is determined from density and speed of sound indirectly using the following formula (eq 2):

2 $${{K_{S,m}^{E}=K}_{S,m}-K_{S,m}^{\mathrm{id}}=K}_{S,m}-\sum_{i}x_{i}K_{S,m,i}-T\sum_{i}x_{i}{E_{p,i}^{2}/C}_{p,i}+T{\left(\sum_{i}x_{i}E_{p,i}\right)}^{2}/\sum_{i}x_{i}C_{p,i}$$

where *K*_*S,m,i*_, *E*_*p,i*_, and *C*_*p,i*_ are the molar isentropic compressibility, the molar isobaric expansion, and the isobaric molar heat capacity of pure component *i*, respectively.

The molar isentropic compressibility (*K*_*S,m*_) is obtained according to eq 3:

3 $$K_{S,m}=-{\left(\frac{\partial V_{m}}{\partial P}\right)}_{S}=V_{m}\frac{1}{u^{2}d}$$

The molar isobaric expansion is estimated using the coefficient of isobaric thermal expansion (*E*_*p*_ = *V*_*m*_α_*p*_), which is calculated from the density data by the expression (eq 4):

4 $$\alpha_{p}=\frac{1}{V_{m}}{\left(\frac{\partial V_{m}}{\partial T}\right)}_{P}=-\frac{1}{d}{\left(\frac{\partial d}{\partial T}\right)}_{P}$$

In this work, the values of isobaric molar heat capacity for water were taken directly from the literature, while the isobaric molar heat capacities of DESs were measured.^31^

The excess molar volumes and excess molar isentropic compressibilities are fitted by the Redlich–Kister polynomial equation (eq 5):^32^

5 $$Y^{E}=x_{1}x_{2}\sum_{i=0}^{2}A_{i}{\left(x_{1}-x_{2}\right)}^{i}$$

where the *A*_*i*_ values are adjustable parameters. These parameters are further used to determine the partial molar volumes (*V̅*_*i*_), which allow a more detailed evaluation of the interactions between the solution components. The symbols *x*_1_ and *x*_2_ denote molar fractions of DES and water, respectively.

The partial molar volumes are calculated using the parameters of Redlich–Kister equation as well as the molar volumes of the pure components (*V*_*i*_^*o*^) by eqs 6 and 7:

6 $${\mathrm{V̅}}_{1}=V_{1}^{o}+{{(x}_{1}-1)}^{2}\sum_{i=0}^{j}A_{i}{\left(2x_{1}-1\right)}^{i}+{{2x_{1}(1-x}_{1})}^{2}\sum_{i=0}^{j}A_{i}{i\left(2x_{1}-1\right)}^{i-1}$$

7 $${\mathrm{V̅}}_{2}=V_{2}^{o}+{x_{1}}^{2}\sum_{i=0}^{j}A_{i}{\left(2x_{1}-1\right)}^{i}-2{x_{1}}^{2}\left(1-x_{1}\right)\sum_{i=0}^{j}A_{i}{i\left(2x_{1}-1\right)}^{i-1}$$

The partial molar volumes at infinite dilution of the solution components are obtained using the following eqs (eqs 8 and 9):

8 $${\mathrm{V̅}}_{1}^{\infty }=V_{1}^{o}+\sum_{i=0}^{j}A_{i}{\left(-1\right)}^{i}$$

9 $${\mathrm{V̅}}_{2}^{\infty }=V_{2}^{o}+\sum_{i=0}^{j}A_{i}$$

### 3.2 Prigogine–Flory–Patterson (PFP) Theory

The Prigogine-Flory-Patterson (PFP) theory, which allows the prediction of excess molar volumes without the need for knowledge of the solution density, was initially developed for systems lacking strong hydrogen bonds or electrostatic interactions.^33–36^ However, it has proven effective in correlating excess molar volumes in more complex mixtures. It has been successfully applied to systems containing ionic liquids and, more recently, to aqueous solutions of deep eutectic solvents.^37,38^ According to this theory, the excess molar volume of a binary solution consists of three contributions: an interactional contribution, a free volume contribution, and a pressure contribution (eq 10):

10 $$\frac{V_{m}^{E}}{x_{1}V_{1}^{*}+x_{2}V_{2}^{*}}=\underset{\left(\mathrm{intr}.\mathrm{contribution}\right)}{\underset{︸}{\frac{({Ṽ}^{1/3}-1){Ṽ}^{2/3}\psi_{1}\Theta_{2}\chi_{12}}{[\left(4/3\right){Ṽ}^{-1/3}-1]P_{1}^{*}}}}+\underset{\left(\mathrm{fv}.\mathrm{contribution}\right)}{\underset{︸}{\frac{-{\left({Ṽ}_{1}-{Ṽ}_{2}\right)}^{2}\left[\left(14/9\right){Ṽ}^{-1/3}-1\right]\psi_{1}\psi_{2}}{[\left(4/3\right){Ṽ}^{-1/3}-1]Ṽ}}}+\underset{\left(P^{*}\mathrm{contribution}\right)}{\underset{︸}{\frac{\left({Ṽ}_{1}-{Ṽ}_{2}\right)\left(P_{1}^{*}-P_{2}^{*}\right)\psi_{1}\psi_{2}}{P_{2}^{*}\psi_{1}+P_{1}^{*}\psi_{2}}}}$$

where V* is the characteristic volume, P* is the characteristic pressure, ψ is the molecular contact energy fraction, θ is the molecular surface fraction, and *Ṽ* is the reduced volume. The calculation of the above parameters is shown in Supporting Information. χ_12_ is the interactional parameter, assumed to be composition independent, and estimated by minimizing the objective function based on the deviations between the experimental and calculated excess volumes, defined as follows (eq 11):

11 $$\mathrm{OF}=\sum_{i=1}^{n}{\left(V_{m,\exp}^{E}-V_{m,\mathrm{calc}}^{E}\right)}^{2}$$

### 3.3 COSMO-RS Computation Methodology

Utilization of the COSMO-RS framework requires, in the first step, the appropriate characteristics of structural diversity of all system constituents.^39^ Since the protocol applied here adheres to previously published schemes, only a brief synopsis is provided here.^40,41^

The conformational analysis of the deep eutectic solvent components was conducted to generate a diverse set of conformers before COSMOtherm (BIOVIA 2024) calculations. Initial molecular structures of the DES constituents, including choline chloride, acetylcholine chloride, propan-1,2-diol, and propan-1,3-diol, were subjected to a systematic conformer search using COSMOconf (BIOVIA 2024). This program employs a combination of molecular mechanics, semiempirical methods, and DFT approaches to explore the conformational space, generating an ensemble of low-energy conformers by systematically varying torsional angles and optimizing geometries. These conformers were then refined using TURBOMOLE (BIOVIA 2024) at the DFT level with the BP86 functional and def2-TZVP basis set, incorporating the COSMO continuum solvation model to account for solvent effects during geometry optimization and energy calculations. The resulting conformers were ranked by their energies, and a representative subset was selected to ensure adequate coverage of the conformational space while minimizing redundancy. Similarly, to capture the structural diversity of molecular contacts, a detailed conformational analysis was conducted to identify the most stable and probable complexes as well. COSMOtherm facilitated the initial generation of molecular pairs by systematically varying their mutual orientations with a 15° rotation step, using the command “CONTACT = {1 2} ssc_probability ssc_weak ssc_ang = 15.0,” which accounted for both hydrogen bonding and weak interactions. The resulting structures, typically far from optimal, were then optimized with TURBOMOLE using the RI-DFT BP86 method. Redundant and high-energy conformers were filtered out based on RMSD and an energy threshold of 2.5 kcal·mol^–1^ relative to the most stable conformer, yielding a refined set of unique contacts for two-molecular complexes. Since COSMOtherm requires conformers optimized in both gas and bulk phases, this process was performed twice. The optimized conformers were converted into “cosmo” and “energy” files compatible with the BP_TZVPD_FINE_21.ctd parameter set for high-accuracy RI-BP/TZVP//TZVPD-FINE calculations in COSMOtherm.

These optimized structures of monomers and pairs were utilized to compute thermodynamic properties and intermolecular interaction energies, such as the concentration-dependent (Δ*G*_*r*(*x*)_ = – *RT* ln *K*_*x*_) and activity-related (Δ*G*_*r*(*a*)_ = – *RT* ln* K*_*a*_) values of Gibbs free energies of bimolecular complexes formation. Both types of values of Gibbs free energy are interrelated due to a fundamental relationship *K*_*a*_ = *K*_*x*_ *K*_γ_, where the latter represents the product of activity coefficients. All these characteristics are directly available in the COSMOtherm output. In computations of all thermodynamic properties, the proportions of HBA and HBD were preserved for the properties represented as experimentally studied systems. This is achieved by explicitly providing mole fractions in the input files of all enumerated components, either in the form of monomers or bimolecular clusters. Hence, despite variations in the water content, the ratio of HBA to HBD was fixed at the experimental value of 1:3.

Furthermore, σ-profiles were generated for all monomeric and dimeric species by analyzing the surface charge densities of the optimized molecular structures. These profiles depict the distribution of screening charges across the molecular surface, serving as a quantitative measure of the molecule’s polarity and hydrogen-bonding tendencies. In COSMOtherm, σ-potential profiles are typically constructed with 61 data points, covering σ values from −0.03 to +0.03 e·Å^–2^ in increments of 0.001 e·Å^–2^. To simplify the parameter set and reduce its potential redundancy, the continuous profile was converted into a step function by averaging every six consecutive σ-potential values, resulting in a condensed 12-point representation. Notably, the charge density range is often divided into three distinct subregions for interpretation: σ in [–0.01 e·Å^–2^, +0.01 e·Å^–2^] corresponds to hydrophobicity (HYD), σ in [+0.01 e·Å^–2^, +0.03 e·Å^–2^] indicates hydrogen bond donor (HBD) capability, and σ within [–0.03 e·Å^–2^, −0.01 e·Å^–2^] reflects hydrogen bond acceptor (HBA) behavior. This segmentation enables both qualitative and quantitative insights into a compound’s chemical nature. In the step-function format, each region is represented by four values, facilitating a streamlined analysis. A similar procedure was already employed for molecular descriptors generation for choline chloride-based DES.^42^

### 3.4 Linear Regression Models Development

To model the density of the aqueous deep eutectic solvents (DESs), a systematic descriptor selection and linear regression approach was employed using Python. Molecular descriptors were derived from concentration-dependent Gibbs free energies, Δ*G*_*r(x)*_, decomposed into enthalpic and entropic contributions Δ*G*_*r*(*x*)_ = Δ*H*_*r*(*x*)_ −*T* · Δ*S*_*r*(*x*)_ alongside σ-potential distributions. These descriptors, augmented with sigma moments and energetic contributions, formed the total pool of 60 descriptors used to characterize all possible homo- and heteromolecular pair interactions within the DES systems. A Python script was developed using the “statsmodels” library for ordinary least-squares (OLS) regression and the “sklearn” library for 5-fold cross-validation.^43,44^ All possible combinations of 1 to 6 descriptors were evaluated, with models assessed based on the adjusted *R* ^2^ and statistical significance (*p*-values <0.01). To manage computational efficiency, a refresh threshold of 5000 models was implemented, retaining only the top 100 models per descriptor count based on adjusted *R* ^2^. The best model was selected from statistically significant candidates, and its applicability domain was analyzed using hat values with a threshold of h* = 3p/*n*, where *p* is the number of parameters and *n* is the number of observations.^45^ Results, including the top models and predictions, were saved for further analysis.

## 4 Results and Discussion

### 4.1 Experimental Density and Speed of Sound of DESs

The density and speed of sound of the studied aqueous solutions of deep eutectic solvents were measured as a function of temperature in the range of 293.15 ÷ 333.15 K at ambient pressure, and their values are included in Tables 4, 5, 6, and 7. Table 3 presents the thermophysical properties of pure DESs, along with available literature data.^46–50^ As can be seen, the experimental values of density for DESs based on choline chloride are in good agreement with the values reported not only by our group but also by different authors. In the case of the speed of sound, the literature only provides data for ChCl:1,2-PG, which differs from ours. However, it is worth noting that the values reported by Dias et al. and Vuksanović et al. also show significant deviations.^46,47^

**Table 3:** Thermophysical Properties of Prepared DESs at *T* = (293.15 to 313.15) K and Atmospheric Pressure (0.1 MPa) and Comparison of Densities and Speeds of Sound with Literature Data^a^

	*d*/(kg·m^–3^)		*u*/(m·s^–1^)			
*T*/K	exptl.	lit.	exptl.	lit.	*C*_*P*_/(J·mol^–1^·K^–1^)	10^4^ α/(K^–1^)
ChCl:1,2-PG						
293.15	1071.83	1071.70^c^	1735.28	1738.9^c^	198.2	5.74
		1071.82^d^ ^,^ ^e^				
298.15	1068.75	1069^b^	1723.09	1743.36^b^	199.6	5.75
		1068.62^c^		1726.1^c^		
		1068.78^d^				
303.15	1065.68	1066^b^	1710.85	1731.04^b^	201.0	5.76
		1065.54^c^		1713.4^c^		
		1065.68^d^				
		1065.71^e^				
308.15	1062.62	1062.47^c^	1698.27	1700.8^c^	203.3	5.77
		1062.61^d^				
313.15	1059.55	1060^b^	1686.05	1705.79^b^	205.6	5.78
		1059.39^c^		1688.1^c^		
		1059.55^d^				
		1059.60^e^				
ChCl:1,3-PG						
293.15	1081.54	1081.96^f^	1822.56		195.4	5.18
		1080.91^e^ ^,^ ^g^				
298.15	1078.74	1079.16^f^	1811.96		197.7	5.18
		1078.14^g^				
303.15	1075.96	1076.37^f^	1801.30		200.0	5.17
		1075.34^e^ ^,^ ^g^				
308.15	1073.18	1073.59^f^	1790.76		202.3	5.16
		1072.55^g^				
313.15	1070.42	1070.82^f^	1776.94		204.6	5.16
		1069.78^e^ ^,^ ^g^				
AChCl:1,2-PG						
293.15	1081.88	1082.0^d^	1696.06		218.0	6.20
298.15	1078.54	1078.7^d^	1682.83		220.1	6.18
303.15	1075.21	1075.4^d^	1669.74		221.6	6.17
308.15	1071.90	1072.1^d^	1656.32		223.2	6.16
313.15	1068.61	1068.8^d^	1642.78		225.7	6.14
AChCl:1,3-PG						
293.15	1093.73	1094.44^f^	1785.41		218.5	5.55
298.15	1090.70	1091.41^f^	1773.85		221.1	5.54
303.15	1087.68	1088.38^f^	1762.30		223.7	5.53
308.15	1084.68	1085.38^f^	1751.20		226.2	5.53
313.15	1081.69	1082.38^f^	1739.67		229.8	5.52

a Standard uncertainties are *u*(*T*) = 0.01 K, *u*(*p*) = 10 kPa, *u*(*d*) = 0.1 kg·m^–3^ and *u*(*u*) = 0.5 m·s^–1^

b Ref 46.

c Ref 47.

d Ref 48.

e Ref 29.

f Ref 49.

g Ref 50.

**Table 4:** Densities, *d*, and Speeds of Sound, *u*, of ChCl:1,2-PG and Water in Their Binary Mixtures at *T* = (293.15 to 313.15) K and Atmospheric Pressure (0.1 MPa)^a^

*T*/K	293.15 K	298.15 K	303.15 K	308.15 K	313.15 K
x_1_	*d*/(kg·m^–3^)				
0.0000	998.20	997.04	995.64	994.03	992.21
0.0494	1018.73	1016.98	1015.07	1013.00	1010.79
0.0984	1034.53	1032.25	1029.86	1027.37	1024.77
0.1903	1053.27	1050.43	1047.52	1044.56	1041.53
0.2855	1063.01	1059.97	1056.89	1053.76	1050.60
0.3790	1067.89	1064.79	1061.65	1058.47	1055.26
0.5061	1070.96	1067.86	1064.74	1061.59	1058.41
0.5982	1071.74	1068.63	1065.51	1062.36	1059.19
0.7007	1072.29	1069.21	1066.12	1063.03	1059.92
0.7886	1072.25	1069.17	1066.10	1063.02	1059.93
0.9072	1072.05	1068.97	1065.89	1062.82	1059.75
0.9453	1071.95	1068.88	1065.80	1062.74	1059.67
1.0000	1071.83	1068.75	1065.68	1062.62	1059.55
x_1_	*u*/(m·s^–1^)				
0.0000	1482.61	1497.21	1509.70	1520.48	1529.39
0.0494	1628.96	1633.95	1637.56	1640.40	1642.20
0.0984	1727.47	1725.64	1722.80	1719.59	1715.80
0.1903	1813.41	1805.18	1797.00	1787.99	1778.75
0.2855	1830.44	1820.44	1809.63	1798.60	1787.42
0.3790	1823.60	1812.53	1801.67	1790.13	1778.44
0.5061	1803.13	1791.59	1780.32	1768.38	1756.41
0.5982	1789.74	1778.06	1766.20	1754.16	1742.07
0.7007	1771.85	1759.95	1747.91	1735.81	1724.07
0.7886	1754.02	1742.08	1730.14	1718.06	1705.96
0.9072	1745.03	1732.67	1720.06	1707.76	1695.57
0.9453	1741.37	1729.20	1716.97	1704.36	1692.13
1.0000	1735.28	1723.09	1710.85	1698.27	1686.05

a Standard uncertainties are *u­(T)* = 0.01 K, *u­(p)* = 10 kPa, *u­(ρ)* = 0.1 kg·m^–3^, *u­(u)* = 0.5 m·s^–1^ and the combined standard uncertainty *u­(x*_1_ *)* = 0.0012

**Table 5:** Densities, *d*, and Speeds of Sound, *u*, of ChCl:1,3-PG and Water in Their Binary Mixtures at *T* = (293.15 to 313.15) K and Atmospheric Pressure (0.1 MPa)^a^

*T*/K	293.15 K	298.15 K	303.15 K	308.15 K	313.15 K
x_1_	*d*/(kg·m^–3^)				
0.0000	998.19	997.03	995.63	994.02	992.21
0.0509	1018.41	1016.70	1014.82	1012.88	1010.69
0.1028	1033.63	1031.59	1029.44	1027.18	1024.82
0.2002	1052.91	1050.43	1047.90	1045.30	1042.65
0.2953	1064.03	1061.37	1058.67	1055.95	1053.18
0.4056	1071.19	1068.46	1065.70	1062.92	1060.12
0.4882	1074.73	1071.97	1069.21	1066.42	1063.62
0.5988	1077.65	1074.88	1072.11	1069.34	1066.55
0.6853	1079.05	1076.27	1073.51	1070.73	1067.95
0.7792	1080.13	1077.35	1074.58	1071.80	1069.03
0.8793	1080.88	1078.09	1075.31	1072.54	1069.77
0.9377	1081.21	1078.32	1075.55	1072.78	1070.03
1.0000	1081.54	1078.74	1075.96	1073.18	1070.42
x_1_	*u*/(m·s^–1^)				
0.0000	1482.420	1497.070	1509.610	1520.340	1529.270
0.0509	1613.680	1619.890	1624.760	1628.730	1631.730
0.1028	1710.540	1710.480	1709.910	1708.760	1706.960
0.2002	1809.010	1803.810	1797.810	1791.310	1784.510
0.2953	1847.960	1839.840	1831.460	1822.770	1813.930
0.4056	1859.270	1850.040	1840.690	1831.080	1820.830
0.4882	1857.890	1848.320	1838.540	1828.250	1818.260
0.5988	1850.350	1840.520	1830.520	1820.340	1810.210
0.6853	1848.850	1838.870	1828.680	1818.520	1808.390
0.7792	1838.680	1828.470	1818.240	1807.960	1797.670
0.8793	1828.530	1818.530	1808.120	1797.640	1787.250
0.9377	1826.120	1814.620	1805.130	1794.450	1783.430
1.0000	1822.560	1811.960	1801.300	1790.760	1776.940

a Standard uncertainties are *u­(T)* = 0.01 K, *u­(p)* = 10 kPa, *u­(ρ)* = 0.1 kg·m^–3^, *u­(u)* = 0.5 m·s^–1^ and the combined standard uncertainty *u­(x*_1_ *)* = 0.0012

**Table 6:** Densities, *d*, and Speeds of Sound, *u*, of AChCl:1,2-PG and Water in Their Binary Mixtures at *T* = (293.15 to 313.15) K and Atmospheric Pressure (0.1 MPa)^a^

*T*/K	293.15 K	298.15 K	303.15 K	308.15 K	313.15 K
x_1_	*d*/(kg·m^–3^)				
0.0000	998.19	997.03	995.63	994.02	992.20
0.0498	1023.41	1021.50	1019.43	1017.20	1014.84
0.0991	1042.13	1039.57	1036.91	1034.15	1031.30
0.1993	1063.25	1060.67	1056.84	1053.56	1050.21
0.2951	1072.80	1069.46	1066.10	1062.70	1059.28
0.3918	1077.53	1074.14	1070.72	1067.26	1063.74
0.4946	1080.09	1076.74	1073.36	1069.98	1066.57
0.5931	1081.39	1077.99	1074.47	1071.02	1067.59
0.6904	1081.87	1078.53	1075.20	1071.85	1068.50
0.7902	1081.98	1078.64	1075.31	1071.98	1068.65
0.8787	1082.08	1078.76	1075.44	1072.13	1068.81
0.9640	1081.83	1078.47	1075.19	1071.89	1068.61
1.0000	1081.88	1078.54	1075.21	1071.90	1068.61
x_1_	*u*/(m·s^–1^)				
0.0000	1482.81	1497.36	1509.94	1520.67	1529.64
0.0498	1640.44	1643.71	1645.89	1647.39	1647.94
0.0991	1736.84	1732.93	1728.52	1723.52	1718.15
0.1993	1807.59	1797.90	1787.87	1777.62	1767.06
0.2951	1813.16	1802.14	1790.33	1778.40	1766.55
0.3918	1788.69	1776.48	1763.96	1751.32	1738.60
0.4946	1767.22	1754.40	1741.46	1728.88	1715.83
0.5931	1751.46	1738.62	1725.65	1712.30	1699.20
0.6904	1731.63	1718.57	1705.37	1692.23	1679.01
0.7902	1716.89	1703.81	1690.61	1677.15	1663.90
0.8787	1705.16	1691.98	1678.77	1665.27	1652.01
0.9640	1696.88	1683.90	1670.04	1656.70	1643.20
1.0000	1696.06	1682.83	1669.74	1656.32	1642.78

a Standard uncertainties are *u­(T)* = 0.01 K, *u­(p)* = 10 kPa, *u­(ρ)* = 0.1 kg·m^–3^, *u­(u)* = 0.5 m·s^–1^ and the combined standard uncertainty *u­(x*_1_ *)* = 0.0012

**Table 7:** Densities, *d*, and Speeds of Sound, *u*, of AChCl:1,3-PG and Water in Their Binary Mixtures at *T* = (293.15 to 313.15) K and Atmospheric Pressure (0.1 MPa)^a^

*T*/K	293.15 K	298.15 K	303.15 K	308.15 K	313.15 K
x_1_	*d*/(kg·m^–3^)				
0.0000	998.19	997.03	995.63	994.02	992.21
0.0498	1022.14	1020.36	1018.43	1016.35	1014.12
0.0997	1040.40	1038.11	1035.71	1033.22	1030.63
0.1955	1062.57	1059.75	1056.89	1053.98	1051.01
0.2923	1075.02	1072.02	1069.16	1066.08	1062.98
0.3902	1082.19	1079.13	1076.05	1072.94	1069.81
0.5041	1087.08	1084.02	1080.95	1077.87	1074.77
0.5958	1089.68	1086.22	1083.16	1080.20	1077.24
0.6948	1091.14	1088.10	1085.05	1082.01	1078.96
0.7852	1092.24	1089.20	1086.17	1083.14	1080.11
0.8801	1093.03	1090.00	1086.97	1083.96	1080.94
0.9661	1093.55	1090.52	1087.50	1084.49	1081.50
1.0000	1093.73	1090.70	1087.68	1084.68	1081.69
x_1_	*u*/(m·s^–1^)				
0.0000	1482.83	1497.43	1509.88	1520.65	1529.59
0.0498	1623.95	1628.79	1632.32	1635.08	1636.88
0.0997	1718.17	1716.53	1714.33	1711.53	1708.05
0.1955	1808.25	1800.96	1792.85	1784.44	1775.82
0.2923	1837.08	1826.99	1816.79	1806.26	1795.71
0.3902	1839.64	1828.82	1817.80	1806.63	1795.47
0.5041	1831.61	1820.39	1809.02	1797.60	1786.17
0.5958	1825.84	1814.02	1802.46	1790.44	1778.86
0.6948	1811.42	1800.51	1789.13	1777.61	1766.15
0.7852	1802.71	1791.30	1780.25	1768.78	1757.40
0.8801	1794.48	1782.98	1771.94	1760.42	1749.04
0.9661	1787.50	1776.02	1764.44	1753.36	1741.99
1.0000	1785.41	1773.85	1762.30	1751.20	1739.67

a Standard uncertainties are *u­(T)* = 0.01 K, *u­(p)* = 10 kPa, *u­(ρ)* = 0.1 kg·m^–3^, *u­(u)* = 0.5 m·s^–1^ and the combined standard uncertainty *u­(x*_1_ *)* = 0.0012

At a fixed temperature, the density of the studied DESs is as follows: AChCl:1,3-PG > AChCl:1,2-PG ≈ ChCl:1,3-PG > ChCl:1,2-PG, while the speed of sound changes along the sequence ChCl:1,3-PG > AChCl:1,3-PG > ChCl:1,2-PG > AChCl:1,2-PG.Thus, both the type of HBA and the structure of the glycol significantly influence the physical properties of DESs. Replacing ChCl with AChCl increases the density of mixtures, probably mainly due to the higher molar mass of AChCl. Similarly, using linear 1,3-propanediol instead of 1,2-propanediol results in a higher density, which can be attributed to stronger hydrogen bonding. Systems based on ChCl exhibit a higher speed of sound compared to those based on AChCl. As with density, the use of 1,3-propanediol in DES results in its higher speed of sound values, which is probably a consequence of a more compact and organized liquid structure.

Adding a small amount of water to a deep eutectic solvent results in a slight increase or decrease in density, depending on the specific mixture. However, beyond a certain water content, the density of all solutions begins to drop significantly. In the case of the speed of sound, a similar trend is observed, but the initial change is more pronounced - for all DES systems, a relatively significant increase in the speed of sound is evident at low water contents. As the water content increases further, the speed of sound typically reaches a maximum and then gradually decreases. Both the density and speed of sound of aqueous DES solutions exceed the ideal value, indicating that intermolecular attractive forces or more favorable mixing behavior result in tighter molecular packing and an organized liquid structure.

For all the DESs studied, both in their pure state and aqueous solutions, the density and speed of sound decrease with increasing temperature due to thermal expansion. An exception of this behavior is observed only in solutions with a small amount of DES (up to 0.05 mole fraction), where an increase in the speed of sound with temperature is noted. This phenomenon can be a result of two competing effects: as the temperature increases, the kinetic energy of the liquid molecules increases, leading to greater movement and, consequently, to an increase in the speed of sound. On the other hand, the decrease in density with increasing temperature has the opposite effect, tending to reduce the speed of sound. Thus, the coexistence of these opposing factors results in nonmonotonous behavior of the speed of sound, which initially (in diluted solutions of DESs) increases with temperature, reaches a maximum and then decreases. Similar findings have been reported in previous studies on aqueous ChCl:malonic acid and ChCl:glutaric acid, as well as in mixtures of polyethylene glycols (e.g., PEG 1000 and PEG 1540).^19,51^ In these systems, the authors also observed the maximum in the temperature dependence of the speed of sound for dilute solutions.

### 4.2 Volumetric Properties

#### 4.2.1 Excess Molar Volumes

The calculated values of excess molar volumes of all studied binary mixtures of DES and water at temperatures between 293.15 and 313.15 K are reported in Tables S1–S4. As a representative example, Figure 1a presents the dependence of *V*_*m*_^*E*^ on the DES mole fraction (*x*_1_) for the ChCl:1,2-PG + water system. Figure 1b depicts analogous plots at 293.15 K for all studied mixtures, comparative data from the literature for choline-based DESs.^28,29^ The dashed lines in both figures correspond to values fitted using the four-parameter Redlich–Kister polynomial equation. The coefficients *A*_*i*_ of the equation were determined using the least-squares method, and they are provided in Table S5, along with their associated root-mean-square deviations (RMSD). As can be seen, the experimental data show excellent agreement with the values *V*_*m*_^*E*^ calculated using the Redlich–Kister equation, as well as with those derived from the density data reported by Aravena et al. for DESs composed of choline chloride and either 1,2-propanediol or 1,3-propanediol.^29^

**Figure 1:**
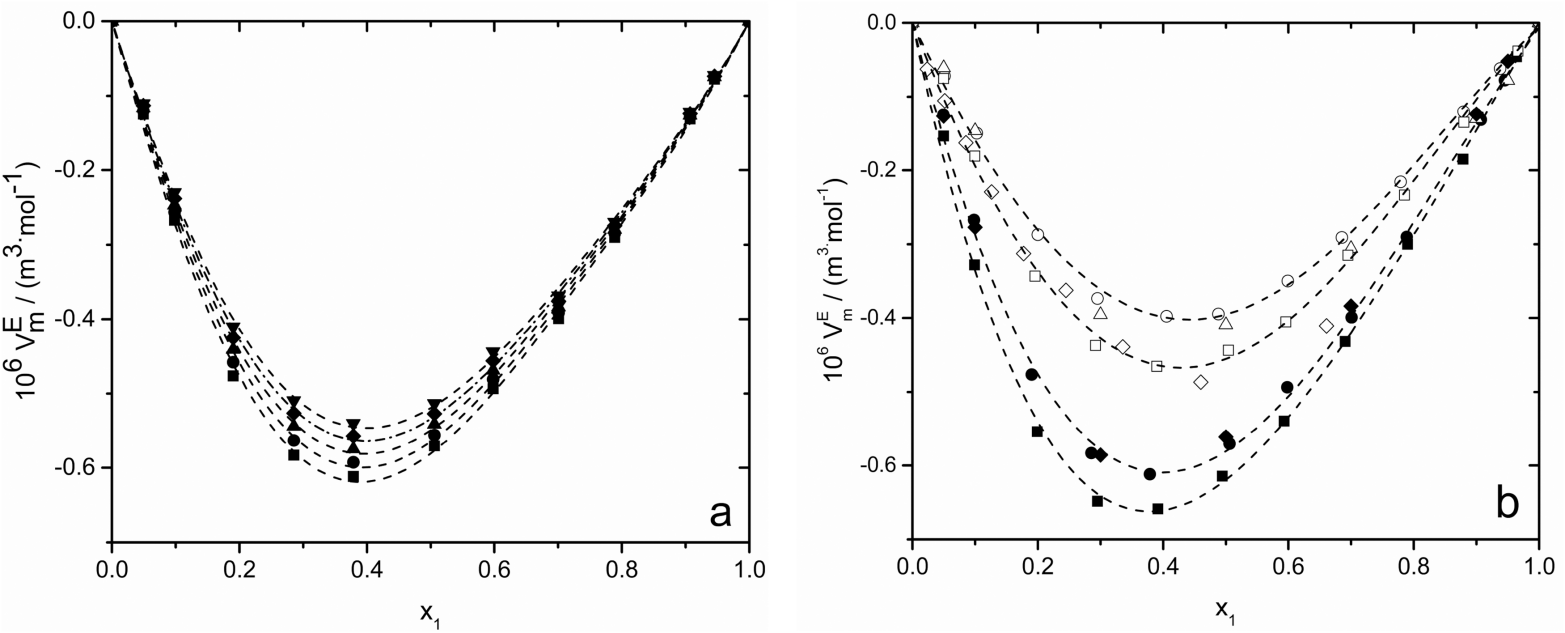
Excess molar volume as a function of mole fraction of DES: (a) for ChCl:1,2-PG at 293.15 K (■); 298.15 K (●); 303.15 K (▲); 308.15 K (⧫); 313.15 K (▼); (b) at 293.15 K for (■) AChCl:1,2-PG; (●) ChCl:1,2-PG; (□) AChCl:1,3-PG; (○) ChCl:1,3-PG; (⧫) ChCl:1,2-PG from ref^29^; (⧫) ChCl:1,3-PG from ref^29^; (◊) ChCl:1,2-PG from ref^28^; dashed line, eq 6.

In contrast, the values of excess molar volumes provided by Zarghampour et al. for aqueous solutions of ChCl:1,2-PG differ significantly, which may be due to the high uncertainty of the density measurements carried out using a pycnometer. This uncertainty is evident even in the reported density value of pure DES (1077 kg·m^–3^), which deviates from the values obtained by other authors (1071.70 or 1071.82 kg·m^–3^).^28,47,48^

The excess molar volume plots reveal asymmetric curves with negative values over the entire composition and temperature range studied. The minima of these curves occur at DES mole fractions around 0.4, indicating a composition where molecular interactions are most favorable for volume contraction. The negative values of *V*_*m*_^*E*^ result from a combination of specific molecular interactions and structural effects. Upon the addition of water to DES, the pre-existing hydrogen bond networks between DES molecules are disrupted. This disruption facilitates the formation of new hydrogen bonds, particularly between water molecules and the chloride anion of the salt, as well as between water and the hydroxyl (−OH) groups of the glycol. Moreover, due to the relatively small size of water molecules compared to those of the DES components, water can occupy the empty spaces in the DES structure. This filling effect increases packing efficiency and leads to a volume contraction, resulting in negative excess molar volumes. The magnitude of these negative values of *V*_*m*_^*E*^ typically increases with the addition of water, up to a specific composition. After reaching a critical water concentration, corresponding to the minimum point of dependence of the excess molar volume on the mole fraction of DES, further addition of water reverses this trend. The excess molar volume starts to increase as the supramolecular structure of DES is gradually disrupted. At this stage, the individual DES components are solvated by water, which weakens the specific DES–DES and DES–water interactions. The packing efficiency also decreases, and as a result, the excess molar volume becomes less negative.

Further analysis of Figure 1b shows that the excess molar volume of the studied deep eutectic solvents changes in the following order: ChCl:1,3-PG > AChCl:1,3-PG > ChCl:1,2-PG > AChCl:1,2-PG. Thus, the observed trend indicates that DESs based on 1,3-propanediol exhibit significantly less negative *V*_*m*_^*E*^ values compared to those based on 1,2-propanediol. This suggests weaker specific interactions between DES and water molecules, probably due to distinctly stronger self-association (DES–DES hydrogen bonding) in the ChCl:1,3-PG and AChCl:1,3-PG systems. The self-association can inhibit the incorporation of water into the DES network, thereby reducing the volume contraction upon mixing. This observation is consistent with the COSMO-RS results (see paragraph 4.5), which showed that 1,3-propanediol has a stronger tendency for self-association than 1,2-propanediol. This stronger internal cohesion in 1,3-PG-based DESs impedes the incorporation of water molecules, resulting in less negative excess molar volumes compared to their 1,2-PG counterparts. The essential effect is the competition between self-association (DES-DES) and cross-association (DES-water). Stronger self-association in 1,3-PG-based DESs makes it harder for water to integrate into the network, leading to less negative excess volumes. The 1,2-PG isomer, with its weaker self-association, allows for more effective mixing and stronger net DES-water interactions, resulting in more negative excess volumes. Moreover, a comparison of *V*_*m*_^*E*^ values for DESs based on the same glycol indicates that mixtures containing acetylcholine chloride as the hydrogen bond acceptor exhibit slightly more negative excess molar volumes than their choline chloride-based counterparts. This behavior can be attributed to the larger quaternary ammonium cation in AChCl, which increases the polarity of the salt and introduces additional hydrogen bonding sites compared to ChCl. These structural features promote stronger intermolecular hydrogen bonding between the AChCl cation and the glycol as confirmed by COSMO-RS calculations. This altered balance of interactions in the pure AChCl DES, compared to the ChCl DES, likely facilitates a more pronounced reorganization and stronger subsequent interactions with water upon mixing, leading to greater volume contraction. The COSMO-RS data provided below confirms that AChCl forms stronger AB heteromolecular pairs with both diols than ChCl does. As a result, the weakening of DES-DES interactions facilitates stronger interactions between DES and water molecules upon mixing. The enhanced interactions between the deep eutectic solvent and water, as well as the more pronounced packing efficiency in the case of larger molecules, result in a greater volume contraction and more negative excess molar volumes. These observations are in agreement with existing literature, which has shown that the molecular structure of both HBA and HBD significantly affects the strength and nature of intermolecular interactions in DES–water systems, thereby influencing volumetric properties and leading to negative *V*_*m*_^*E*^.^52,53^

The increase in excess molar volume with rising temperature for all studied systems suggests that specific intermolecular interactions predominantly govern the volumetric behavior of aqueous solutions of glycol-based deep eutectic solvents. Similar trends have been reported by other authors for aqueous mixtures of carboxylic acid–based DESs.^18–22^ This behavior is commonly attributed to the temperature-induced weakening of hydrogen bonding and other specific interactions, which outweighs any enhancement in molecular packing due to increased thermal motion. As a result, the disruption of structured associations between unlike components leads to an expansion in molar volume with temperature.

#### 4.2.2 Partial Molar Volumes

The partial molar volumes of DES and water *V̅*_*i*_ were calculated using the parameters of the Redlich–Kister equation, as outlined in eqs 6 and 7. Table S7 includes their values at different mole fractions of DES for each temperature. Figure 2 shows the graphical behavior of excess partial molar volumes of DES and water for all studied mixtures at 293.15 K, calculated using the relation: *V̅*_*i*_^*E*^ = *V̅*_*i*_ – *V*_*i*_^*o*^. As a representative example, Figure 3 presents the temperature dependence of the excess partial molar volumes of DES and water in the ChCl:1,2-PG system. The excess partial molar volumes at infinite dilution, which provide direct information on solvation in mixtures, were estimated from the partial molar volumes at infinite dilution. At the same time, the latter were calculated according to eqs 8 and 9 and are reported in Table 8 for all temperatures studied.

**Figure 2:**
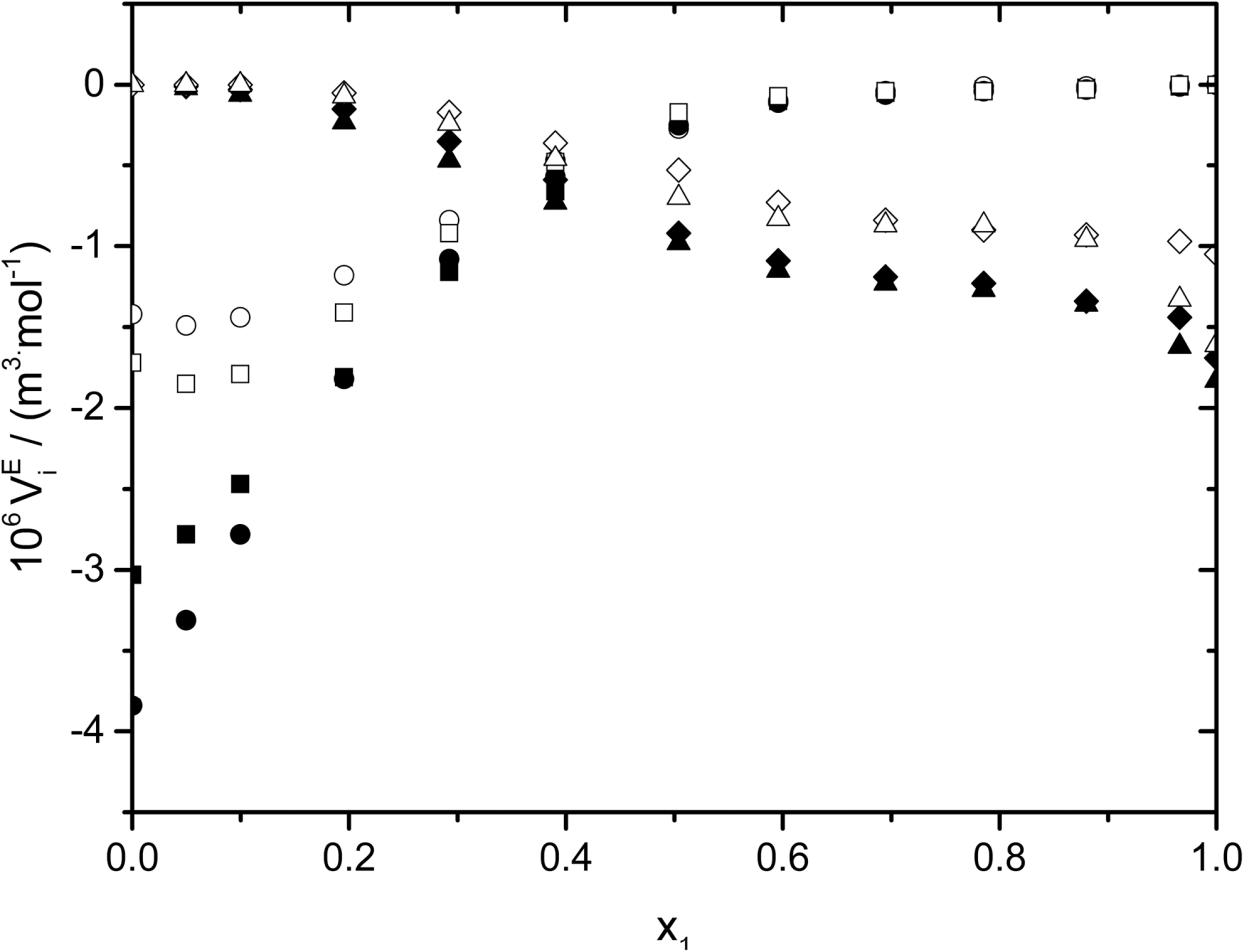
Excess partial molar volumes of DESs and water as a function of mole fraction of DES at 293.15 K: (■) AChCl in aq. AChCl:1,2-PG; (●) ChCl in aq. ChCl:1,2-PG; (□) AChCl in aq. AChCl:1,3-PG; (○) ChCl in aq. ChCl:1,3-PG; water in (▲) aq. AChCl:1,2-PG; (⧫) aq. ChCl:1,2-PG; (Δ) aq. AChCl:1,3-PG; (◊) aq. ChCl:1,3-PG;; (⧫).

**Figure 3:**
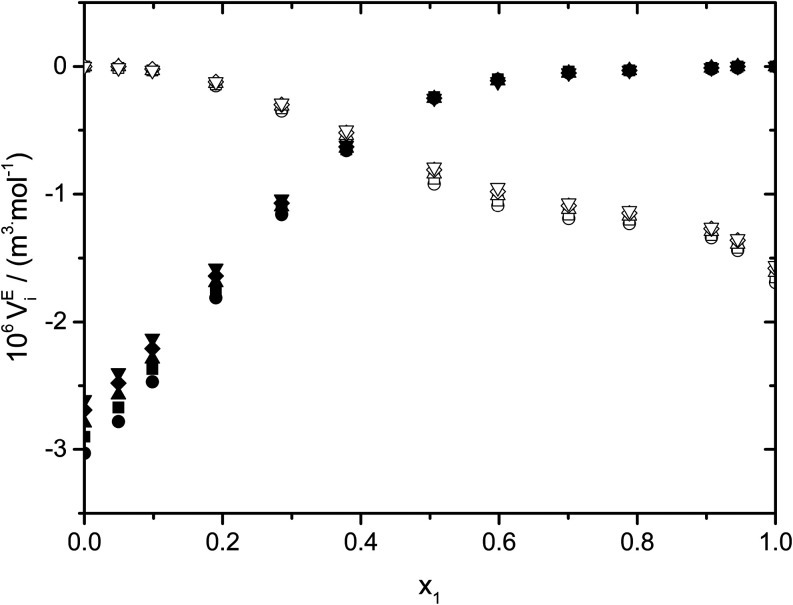
Excess partial molar volumes of DESs and water as a function of mole fraction of ChCl:1,2-PG: (●) 293.15 K; (■) 298.15 K; (▲) 303.15 K; (⧫) 308.15 K; (▼) 313.15 K; full symbols - ChCl:1,2-PG; open symbols – water.

**Table 8:** Excess Partial Molar Volumes at Infinite Dilution, *V̅*_*i*_^*E*, ∞^, of DESs and Water in Their Binary Mixtures at *T* = (293.15 to 313.15) K and Atmospheric Pressure (0.1 MPa)

	10^6^ *V̅*_1_^*E*,∞^/(m^3^·mol^–1^)	10^6^ *V̅*_2_^*E*,∞^/(m^3^·mol^–1^)	10^6^ *V̅*_1_^*E*,∞^/(m^3^·mol^–1^)	10^6^ *V̅*_2_^*E*,∞^/(m^3^·mol^–1^)	10^6^ *V̅*_1_^*E*,∞^/(m^3^·mol^–1^)	10^6^ *V̅*_2_^*E*,∞^/(m^3^·mol^–1^)	10^6^ *V̅*_1_^*E*,∞^/(m^3^·mol^–1^)	10^6^ *V̅*_2_^*E*,∞^/(m^3^·mol^–1^)
*T*/K	ChCl:1,2-PG (1) + water (2)		ChCl:1,3-PG (1) + water (2)		AChCl:1,2-PG (1) + water (2)		AChCl:1,3-PG (1) + water (2)	
293.15	–3.03	–1.69	–1.42	–1.05	–3.83	–1.82	–1.73	–1.61
298.15	–2.90	–1.65	–1.33	–1.02	–3.74	–1.82	–1.59	–1.56
303.15	–2.79	–1.61	–1.26	–1.01	–3.47	–1.81	–1.46	–1.55
308.15	–2.70	–1.58	–1.20	–0.99	–3.32	–1.80	–1.36	–1.51
313.15	–2.61	–1.56	–1.15	–0.98	–3.18	–1.77	–1.35	–1.24

As shown in Figure 2 and Table 8, the excess partial molar volumes, including those for infinite dilution, are negative for both DES and water in each of the studied systems. Because the solute–solute interaction disappears at infinite dilution, the partial molar volumes at infinite dilution are a direct source of information about the solute–solvent interaction. The obtained results therefore indicate preferential solvation of each component by the other, which is consistent with literature reports on hydrogen-bonded systems involving deep eutectic solvents and water.^54,55^ A more negative value of *V̅*_*i*_^*E*^ is generally associated with stronger specific interactions, particularly hydrogen bonding. The obtained results reveal that both 1,2-propanediol- and acetylcholine chloride-based DESs exhibit more pronounced negative excess partial molar volumes compared to those containing 1,3-propanediol or choline chloride, suggesting stronger interactions with water molecules in these systems. This effect is especially evident in dilute DES solutions (*x*_1_ < 0.4), where the DES structure is disrupted, and individual DES components become available for hydrogen bonding with water. While both 1,3-propanediol and acetylcholine chloride form more stable hydrogen bond networks due to their symmetrical molecular structures and the presence of additional hydrogen bonding sites, respectively, the 1,2-propanediol and choline chloride structures facilitate more effective interactions with surrounding water molecules, resulting in enhanced solvation. The excess partial molar volumes of water change according to the sequence ChCl:1,3-PG > AChCl:1,3-PG > ChCl:1,2-PG > AChCl:1,2-PG, confirming these findings. Moreover, in solutions with a DES mole fraction higher than about 0.4, the values of *V̅*_*i*_^*E*^ of water are more negative than those of DES. It can therefore be suspected that in such compositions, the hydrogen bond structure is organized in a manner where water molecules occupy central positions, surrounded by the DES.

Further analysis of Table 8 reveals that the excess partial molar volumes at infinite dilution for 1,2-propanediol-based DESs are significantly more negative than those observed for water over the entire temperature range studied. In contrast, in the systems containing 1,3-propanediol, the excess partial molar volumes of DESs at infinite dilution are also negative but much closer in value to those for water. Thus, the obtained results indicate that in systems with 1,2-propanediol, DES molecules are more effectively solvated by water. In systems with 1,3-propanediol, solvation is still present, but the solvation environment is more balanced, where both types of solvation structures—DES molecules solvated by water and water molecules solvated by DES—form with comparable ease.

Figure 3 shows that the partial excess molar volumes of DES and water increase with increasing temperature. This trend is attributed to the weakening of hydrogen bonding interactions within the system as thermal energy increases. It is consistent with previously reported thermodynamic studies of hydrogen-bonded solvent systems, where a temperature-dependent expansion due to reduced intermolecular cohesion is commonly observed.^52,53^

### 4.3 PFP Theory

The PFP model was applied as introduced by eq 10. All required pure component parameter values for this model, at each investigated temperature and for all studied systems, are presented in Table S8 of the Supporting Information. These parameters were calculated using equations provided in the SI. Among them, only the coefficient of isobaric thermal expansion (α_i_) for both the DESs and water was determined from the temperature dependence of density according to eq 4.

The values of the three contributions of the PFP theory: the interaction (*V*_*int*_^*E*^), free volume (*V*_*fv*_^*E*^), and internal pressure terms (*V*_P*_^E^) and the interactional parameter (χ_12_) at *x*_1_ = 0.4 along with root-mean-square deviations (RMSD) are reported in Table 9 for each of the investigated systems.

**Table 9:** Calculated Values of the Interactional Parameter, χ_12_, Root Mean Square Deviations, RMSD, and the Three Contributions (*V*_*int*_^*E*^, *V*_*fv*_^*E*^, *V*_P*_^E^) from the PFP Theory to the Excess Molar Volumes for the Binary Mixtures of Deep Eutectic Solvents with Water at *x*_*1*_ = 0.4 and *T* = (293.15/313.15) K

*T*/K	293.15	298.15	303.15	308.15	313.15	293.15	298.15	303.15	308.15	313.15
	ChCl:1,2-PG (1) + water (2)					ChCl:1,3-PG (1) + water (2)				
10^6^ χ_12_/(J·m^–3^)	–329.21	–287.34	–249.23	–223.76	–194.66	–275.95	–231.55	–192.10	–164.61	–138.46
10^6^ *V*_*int*_^*E*^/(m^3^·mol^–1^)	–1.255	–1.080	–0.925	–0.805	–0.695	–0.951	–0.785	–0.642	–0.544	–0.456
10^6^ *V*_P*_^E^/(m^3^·mol^–1^)	0.636	0.502	0.382	0.292	0.201	0.555	0.422	0.306	0.208	0.128
10^6^ *V*_*fv*_^*E*^/(m^3^·mol^–1^)	–0.033	–0.032	–0.029	–0.022	–0.017	–0.027	–0.024	–0.019	–0.014	–0.009
10^6^ RMSD/(m^3^·mol^–1^)	0.033	0.015	0.010	0.017	0.025	0.024	0.012	0.021	0.028	0.036
	AChCl:1,2-PG (1) + water (2)					AChCl:1,3-PG (1) + water (2)				
10^6^ χ_12_/(J·m^–3^)	–331.91	–301.51	–250.47	–224.66	–195.49	–295.29	–249.82	–209.20	–173.46	–156.25
10^6^ *V*_*int*_^*E*^/(m^3^·mol^–1^)	–1.390	–1.244	–1.020	–0.906	–0.783	–1.101	–0.922	–0.762	–0.625	–0.559
10^6^ *V*_P*_^E^/(m^3^·mol^–1^)	0.759	0.603	0.464	0.342	0.238	0.643	0.501	0.375	0.267	0.177
10^6^ *V*_*fv*_^*E*^/(m^3^·mol^–1^)	–0.044	–0.042	–0.038	–0.033	–0.025	–0.032	–0.030	–0.025	–0.020	–0.014
10^6^ RMSD/(m^3^·mol^–1^)	0.048	0.039	0.025	0.010	0.010	0.030	0.011	0.013	0.027	0.033

As can be seen, the RMSD values, ranging from (0.010 to 0.048) 10^–6^ m^3^·mol^–1^, indicate that the PFP theory satisfactorily predicts the excess molar volumes of the DES systems studied. Despite the PFP model’s inability to account for strong interactions between components, such as electrostatic and hydrogen bonding, its agreement with experimental data is a strong indicator of its accuracy in reproducing the volumetric behavior of the studied mixtures. It is worth noting that this level of accuracy was achieved using only one adjustable parameter, which highlights the model’s ability to reproduce key features of excess molar volume profiles in these types of complex systems.

In all of the studied DES-water mixtures, both the interaction and free volume contribution are negative, while the pressure contribution is positive. However, the magnitude of the free volume and pressure contributions are significantly smaller compared to the interaction term. Thus, the obtained results indicate that the interaction contribution dominates the overall behavior of excess molar volume, determining both its sign and magnitude in all systems and temperature ranges studied. This conclusion is consistent with the findings on the temperature dependence of excess molar volume, which suggests that the packing effect is of minor importance for the systems studied. In the studied systems, water molecules preferentially engage in strong, specific interactions with DESs, leading to a reorganization of the hydrogen bond network rather than the creation of additional void space. The introduction of water therefore promotes local structural rearrangement and, in some cases, more efficient packing, so that any interstitial accommodation of water molecules in the voids of the DES structure is largely compensated by increased intermolecular interactions. As a result, the excess molar volume is dominated by interaction contributions rather than by changes in free volume.

The positive pressure contribution supports the hypothesis that the structural breakdown of the DES network during mixing and the formation of specific interactions, such as hydrogen bonds, between DES and water. The values of the interaction parameter (χ_12_), which is involved in the interaction contribution of the PFP theory and represents the binary interaction of the two components in the mixture, are negative for all systems studied. Thus, they also confirm that DES–water interactions are more favorable than the self-association in pure DES or pure water, indicating strong specific interactions upon mixing. Furthermore, increasing temperature results in smaller negative values of the interaction parameter, indicating a decrease in the strength of DES–water hydrogen bonds with increasing thermal energy. This temperature dependence further supports the conclusion that specific interactions, rather than packing or free volume effects, determine the excess molar volume behavior in the studied DES–water systems. Since the absolute values of the interaction, free volume, and interaction parameter follow the order: ChCl:1,3-PG < AChCl:1,3-PG < ChCl:1,2-PG < AChCl:1,2-PG, it can be concluded that DESs-based on 1,2-propanediol, especially those containing acetylcholine chloride (AChCl), exhibit the strongest interactions with water, likely due to more favorable hydrogen bond geometry. This finding is in agreement with the earlier descriptions of the volumetric properties of the studied systems.

### 4.4 Acoustic Properties

Tables S1–S4 in the Supporting Information present the calculated values of excess molar isentropic compressibility for aqueous solutions of the studied DESs in the entire range of compositions at temperatures ranging from 293.15 to 313.15 K. Figure 4a shows the temperature dependence of excess isentropic compressibility for the system (ChCl:1,2-PG + water), as an example. Figure 4b depicts the plots of *K*_*S*,*m*_^*E*^ as a function of the DES mole fraction for all studied mixtures at 293.15 K. It is evident that these dependences are even more asymmetric than those observed for the excess molar volume, and their minima occur almost at a DES mole fraction of 0.3. The dashed lines in the figures connect the experimental data points well, indicating that the Redlich–Kister equation provides a practical and reliable approach for estimating the excess molar isentropic compressibility in the entire concentration range. The estimated parameters of the equation are presented in Table S6.

**Figure 4:**
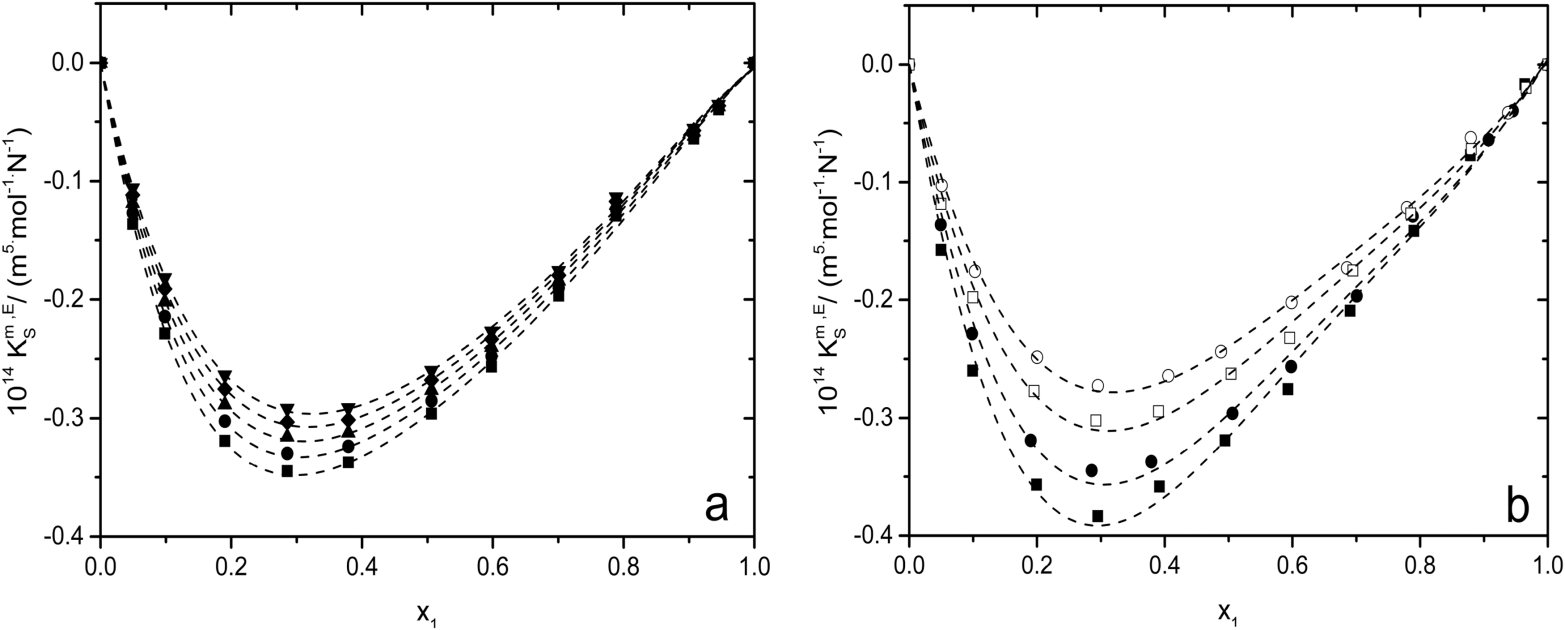
Excess molar isentropic compressibility as a function of mole fraction of DES: (a) for ChCl:1,2-PG at 293.15 K (■); 298.15 K (●); 303.15 K (▲); 308.15 K (⧫); 313.15 K (▼); (b) at 293.15 K for (■) AChCl:1,2-PG; (●) ChCl:1,2-PG; (□) AChCl:1,3-PG; (○) ChCl:1,3-PG; dashed line, eq 6.

The excess isentropic molar compressibility in all studied systems is negative regardless DES composition and temperature. This means that investigated solutions are less compressible than their corresponding ideal mixtures. This effect is attributed to molecular packing and stronger intermolecular interactions between the unlike components of the solution. The order of excess isentropic compressibility values is as follows: AChCl:1,2-PG < ChCl:1,2-PG < AChCl:1,3-PG < ChCl:1,3-PG. Thus, it corresponds to the observed sequence of excess molar volume, confirming that both thermodynamic parameters indicate strong interactions between water and DES molecules, particularly pronounced in 1,2-propanediol-based DES solutions. Moreover, as shown in Figure 4a, the excess molar isentropic compressibility values become less negative with increasing temperature in all studied systems. This indicates a weakening of intermolecular interactions, as also was suggested by the analysis of volumetric properties.

### 4.5 Intermolecular Interactions between DES Components via COSMO-RS

Rational representation of components’ structural diversities was done by including up to ten of the most probable conformation as described in the methodology section. Tables 10, 11, and 12 compile representative geometries with their corresponding charge density distributions. The mutual affinities of the investigated DES constituents were determined using the standard methodology for thermodynamic properties computations, which was stated in terms of the values of the Gibbs free energy of all potential combinations of pair formation between DES components. Based on their room-temperature Gibbs free energy values, the examination of intermolecular interactions in aqueous deep eutectic solvents shows clear trends in the homomolecular (AA, BB) and heteromolecular (AB, AW, BW) contacts. The choline chloride-based AA interactions in ChCl:1,2-PG and ChCl:1,3-PG for the homomolecular pairs show identical affinities of −10.54 kJ·mol^–1^, indicating that the self-interaction of the choline chloride component is unaffected by the type of propanediol used (1,2-propanediol vs 1,3-propanediol). However, the AChCl:1,2-PG and AChCl:1,3-PG exhibit noticeably higher AA affinities at −15.21 kJ·mol^–1^, suggesting that the homomolecular cohesion within the DES structure is improved by substituting acetylcholine chloride for choline chloride. All systems exhibit the same pattern for the BB interactions, which stand in for the propanediol self-interactions: −13.30 kJ·mol^–1^ for 1,2-propanediol (ChCl:1,2-PG and AChCl:1,2-PG) and a stronger −14.72 kJ·mol^–1^ for 1,3-propanediol (ChCl:1,3-PG and AChCl:1,3-PG). This implies that because of variations in molecule shape or hydrogen-bonding properties, 1,3-propanediol creates more advantageous homomolecular interactions than 1,2-propanediol. Regarding the heteromolecular interactions, the AB affinity is −11.60 kJ·mol^–1^ in ChCl:1,2-PG and rises to −13.32 kJ·mol^–1^ in ChCl:1,3-PG, demonstrating the improved compatibility of choline chloride with 1,3-propanediol. This pattern is more pronounced in systems based on acetylcholine chloride, where the highest AB interaction is shown between AChCl:1,2-PG and AChCl:1,3-PG, with AChCl:1,2-PG exhibiting an AB affinity of −13.68 kJ·mol^–1^ and AChCl:1,3-PG reaching −15.06 kJ·mol^–1^. Based on these findings, 1,3-propanediol regularly outperforms 1,2-propanediol, suggesting that acetylcholine chloride creates more advantageous heteromolecular interactions with propanediols than choline chloride. The salt and hydrogen-bond donor work in concert to enhance the stability of the DES, as evidenced by the order of AB affinities (AChCl:1,3-PG > AChCl:1,2-PG > ChCl:1,3-PG > ChCl:1,2-PG).

**Table 10:**
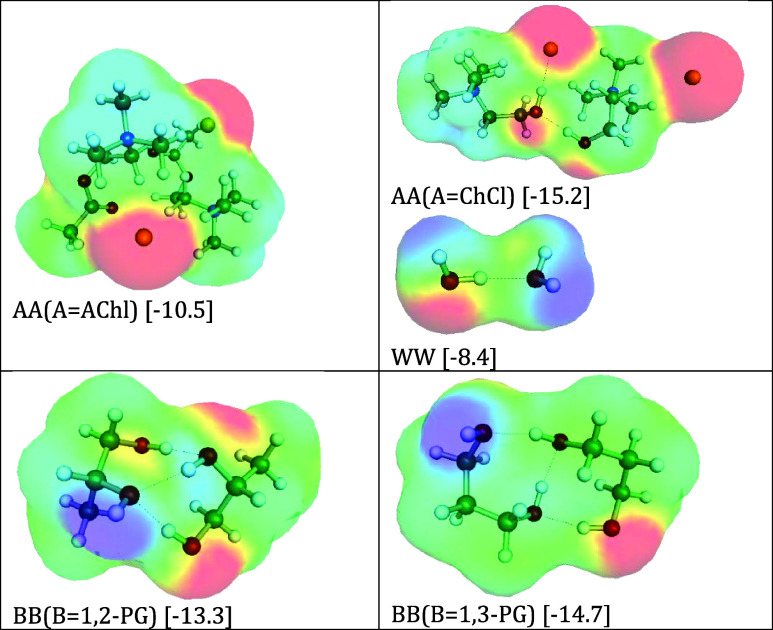
Representative Structures of the Most Probable Homo-Molecular Pairs Formed between Studied DESs Constituents^a^

a The values represent concentration independent Gibbs free energies (in kJ·mol^–1^) computed based on activity values determined using COSMOtherm at room temperature.

**Table 11:**
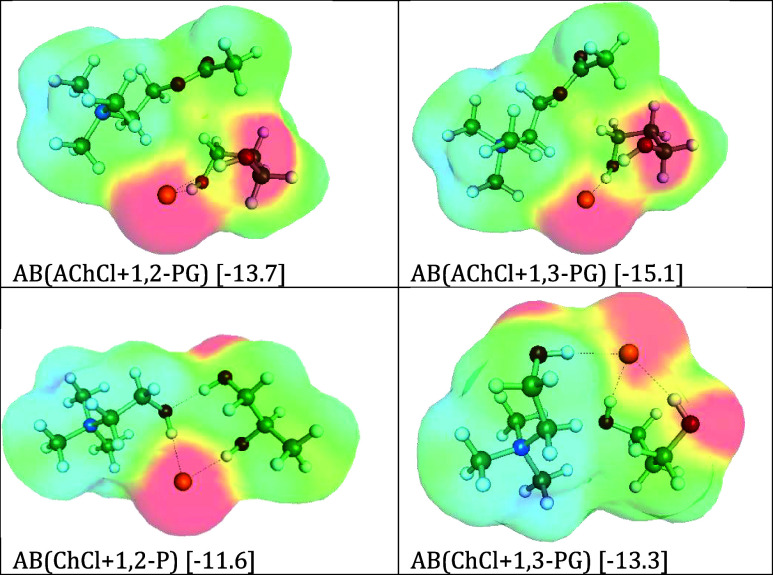
Representative Structures of the Most Likely Hetero-Molecular Pairs That Formed between the DES Ingredients under Study^a^

a The supplied values are concentration independent Gibbs free energies (in kJ·mol^–1^) calculated from activity values at room temperature.

**Table 12:**
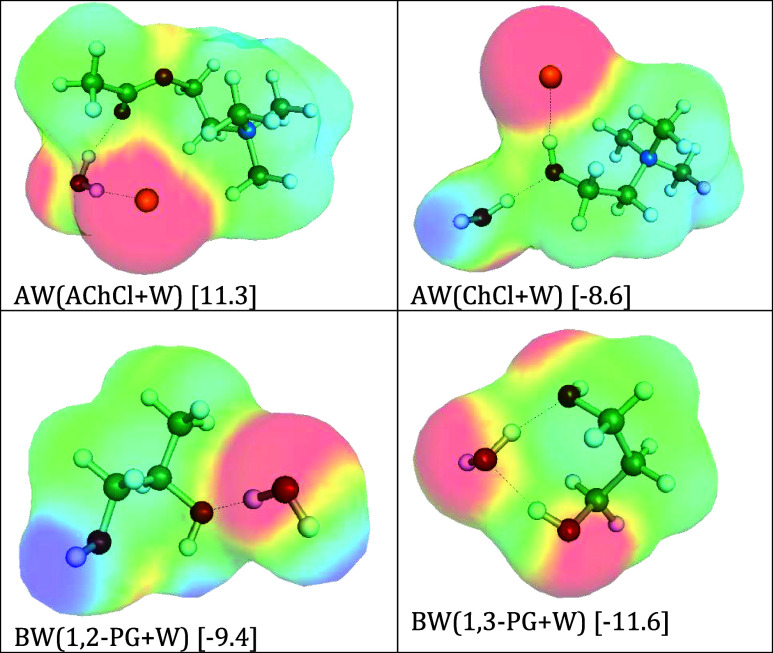
Representative Structures of the Most Probable Hetero-Molecular Pairs Formed Between studied DES Constituents and Water^a^

a The values represent concentration independent Gibbs free energies (in kJ·mol^–1^) computed based on activity values determined using COSMOtherm at room temperature.

Additional information about the solvation behavior of these DESs can be gained from interactions with water (AW and BW pairings). The choline chloride systems (ChCl:1,2-PG and ChCl:1,3-PG) have significantly weaker AW affinities (which represent interactions between the chloride salt and water) at −8.56 kJ·mol^–1^ than the cholinergic chloride systems (AChCl:1,2-PG and AChCl:1,3-PG), which had AW affinities of −11.31 kJ·mol^–1^. Because of its increased polarity or hydrogen-bonding sites, acetylcholine chloride appears to have a greater attraction to water. In contrast, the BW affinities, which represent interactions between glycol and water, are −9.41 kJ·mol^–1^ for 1,2-propanediol (ChCl:1,2-PG and AChCl:1,2-PG) and −11.63 kJ·mol^–1^ for 1,3-propanediol (ChCl:1,3-PG and AChCl:1,3-PG), which are consistent with the BB trends. This supports the finding that 1,3-propanediol interacts with things more strongly than 1,2-propanediol, both with water and with itself. The baseline for water–water interactions, which seem to be independent of the DES composition, is the constant WW affinity of −8.40 kJ·mol^–1^ in all systems.

These concentration profiles provide a direct link to the observed volumetric trends, such as the excess molar volumes. The more negative *V*_*m*_^*E*^ values in 1,3-PG DESs (e.g., ChCl:1,3-PG showing the largest magnitude) align with their stronger AB heteromolecular affinities (−13.32 to −15.06 kJ·mol^–1^), which enhance unlike-molecule interactions and promote greater volume contraction upon aqueous mixing. Conversely, the less negative *V*_*m*_^*E*^ in 1,2-PG systems correlates with weaker AB affinities (–11.60 to −13.68 kJ·mol^–1^), resulting in reduced mixing efficiency despite comparable or slightly weaker BB self-association.

### 4.6 COSMO-RS Speciation Analysis

To move beyond pairwise affinities and quantify the equilibrium distribution of molecular species in the aqueous DES mixtures, a speciation model based on concentration-dependent equilibrium constants (*K*_*x,ij*_) was developed, keeping in mind that *K*_*a*_ = *K*_*x*_ · *K*_γ_. The model considers all possible homomolecular (AA, BB, WW) and heteromolecular (AB, AW, BW) pairs, alongside the monomeric species (A, B, W). The equilibrium for the formation of a pair *ij* from monomers *i* and *j* is defined simply as *i + j = ij*, with the concentration-dependent equilibrium constant given by *K*_*x*_ *= x*_*ij*_ */(x*_*i*_ *·x*_*j*_ *)*, where where *x*_*i*_, *x*_*j*_, *x*_*ij*_ are the equilibrium mole fractions of the monomers and the pair, respectively. The total amount of each component in the system is conserved. This leads to a set of mass balance equations that relate the initial, total mole fractions of the monomers (*a, b, w*) to the equilibrium mole fractions of all species containing that component. The resulting system of coupled, nonlinear equations is

12 $$\begin{array}{l} a=x_{A}+2K_{\mathrm{AA}}\cdot x_{A}^{2}+K_{\mathrm{AB}}\cdot x_{A}\cdot x_{A}+K_{\mathrm{AW}}\cdot x_{A}\cdot x_{W}\\ b=x_{B}+2K_{\mathrm{BB}}\cdot x_{B}^{2}+K_{\mathrm{AB}}\cdot x_{A}\cdot x_{B}+K_{\mathrm{BW}}\cdot x_{B}\cdot x_{W}\\ w=x_{W}+2K_{\mathrm{WW}}\cdot x_{W}^{2}+K_{\mathrm{AW}}\cdot x_{A}\cdot x_{W}+K_{\mathrm{BW}}\cdot x_{B}\cdot x_{W} \end{array}$$

where in eq 12, subscripts stands for *A* = HBA, *B* = HBD, and *W* = water, *a*, *b*, and *w* are the initial mole of monomers. The values of mole fractions in the equilibrium state are presented by *x*_*i*_ (AA,BB,WW, for homomolecular pairs, AB,AW,BW for heteromolecular pairs and A,B,W for monomeric forms). This system of equations cannot be solved analytically. Therefore, a numerical procedure was implemented in Python using the scipy.optimize.root function with the fsolve method. The objective was to find the roots (*x*_*A*_, *x*_*B*_, *x*_*W*_, *x*_*A*_, *x*_*B*_, *x*_*W*_) that minimize the residuals of the mass balance equations. The code takes as input the matrix of *K*_*x,ij*_ values for all systems and compositions and returns the complete set of equilibrium mole fractions for all species.

The obtained values reveal interesting features of studied DES by providing valuable insights into the distribution and interaction behavior of the components as a function of the overall DES mole fraction in the aqueous mixtures. These profiles plotted in Figure 5, expressed as mole fractions (*x*_*i*_) of the homomolecular (*i* = AA, BB, WW) and heteromolecular (*i* = AB, AW, BW) pairs, reveal distinct differences in the molecular organization and interaction preferences across the four DES systems. This can be attributed to the variations in their chemical compositions - choline chloride versus acetylcholine chloride as the salt, and 1,2-propanediol versus 1,3-propanediol as the hydrogen-bond donor. The fact that in all cases water has dominant concentration in almost whole range of concentrations is not surprising. However, due to strong dimerization affinity, in the pairwise additive solution model, water is represented mostly by dimers. This indicates that water molecules preferentially self-associate in dilute DES conditions, consistent with the relatively weak interactions of water with the DES components (AW and BW affinities of −8.56 and −9.41 kJ·mol^–1^, respectively).

**Figure 5:**
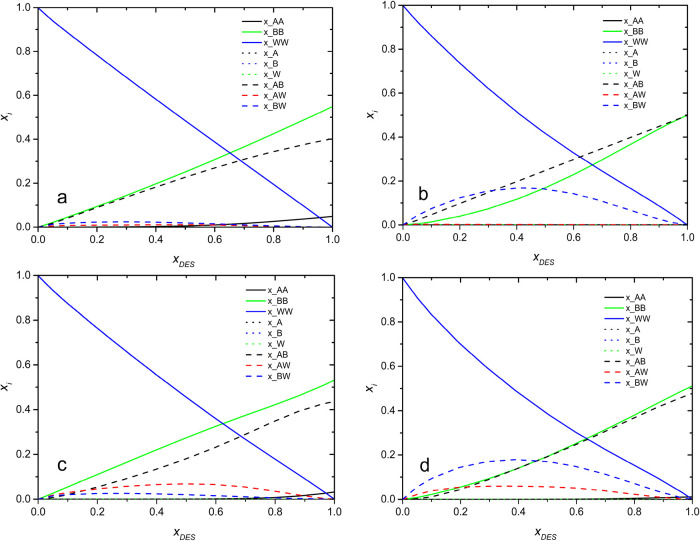
Concentration profiles of four studied types of DES: (a) ChCl:1,2-PG, (b) ChCl:1,3-PG, (c) AChCl:1,2-PG, (d) ChCl:1,3-PG at room temperature determined based on concentration dependent equilibrium constant, *Kx*, defining affinity toward every combination of pair formation. Key trends include dominant WW (water–water) dimerization across all systems, reflecting weak solvation of DES components by water; stronger BB (HBD self-association) in 1,3-PG systems (peaking at ∼0.7–0.8) compared to 1,2-PG (∼0.6), due to enhanced hydrogen-bonding in 1,3-propanediol; and more pronounced AB (HBA-HBD) heteroassociations in AChCl-based DESs (up to ∼ 0.5), particularly with 1,3-PG, indicating synergistic salt-diol compatibility.

The *x*_*BB*_ interactions rise steadily from near 0 to about 0.6 for dry ChCl:1,2-PG (Figure 5a), reflecting the increasing concentration of 1,2-propanediol dimers and its strong self-affinity (−13.30 kJ·mol^–1^). The *x*_*AA*_ (choline chloride dimers) and *x*_*AB*_ (choline chloride-1,2-propanediol) remain relatively low across the concentration range, peaking at around 0.4 and 0.3, respectively, which aligns with their less favorable Gibbs free energies (−10.54 kJ·mol^–1^ for AA and −11.60 kJ·mol^–1^ for AB). The AW and BW pairs show the lowest concentrations, remaining below 0.2, underscoring the limited solvation of DES components by water in this system. In ChCl:1,3-PG (choline chloride +1,3-propanediol), the concentration profile (Figure 5b) exhibits similar trends to ChCl:1,2-PG but with notable differences driven by the change in 1,3-propanediol. The *x*_*BB*_ systematically rises to around 0.7, which is higher than in ChCl:1,2-PG. This can be attributed to the stronger self-affinity of 1,3-propanediol (−14.72 kJ·mol^–1^) compared to 1,2-propanediol, leading to a greater propensity for this diol to self-association. The *x*_*AB*_ profile also increases more significantly, reaching approximately 0.4, consistent with the enhanced AB affinity (−13.32 kJ·mol^–1^) due to better compatibility between choline chloride and 1,3-propanediol. The *x*_*AA*_ profile remains comparable to ChCl:1,2-PG at around 0.4, as the AA affinity is unchanged (−10.54 kJ·mol^–1^). The *x*_*BW*_ profile shows a slight increase compared to ChCl:1,2-PG, peaking near 0.2, reflecting the stronger BW affinity (−11.63 kJ·mol^–1^) with 1,3-propanediol, while *x*_*AW*_ remains low, similar to ChCl:1,2-PG.

Moving to AChCl:1,2-PG (acetylcholine chloride +1,2-propanediol), the concentration profile (Figure 5c) reveals distinct differences due to the consequences of substitution of choline chloride with acetylcholine chloride on the components mutual affinities. The *x*_*AA*_ profile rises prominently to around 0.5 at pure DES, higher than in ChCl:1,2-PG and ChCl:1,3-PG. This reflects the stronger self-affinity of acetylcholine chloride (−15.21 kJ·mol^–1^) compared to choline chloride, leading to greater clustering of the salt molecules. The *x*_*BB*_ profile mirrors ChCl:1,2-PG, reaching about 0.6, as the 1,2-propanediol component is the same. However, the *x*_*AB*_ profile increases to around 0.4, higher than in ChCl:1,2-PG, consistent with the stronger AB affinity (−13.68 kJ·mol^–1^) due to improved interactions between acetylcholine chloride and 1,2-propanediol. The *x*_*AW*_ profile also shows a noticeable increase, peaking near 0.2, which aligns with the enhanced AW affinity (−11.31 kJ·mol^–1^) of acetylcholine chloride with water, while *x*_*BW*_ remains similar to ChCl:1,2-PG at around 0.1.

Finally, AChCl:1,3-PG (acetylcholine chloride +1,3-propanediol) exhibits the most pronounced differences (Figure 5d). The *x*_*BB*_ profile rises sharply to nearly 0.8 for pure DES, the highest among all DESs, reflecting the combined effect of 1,3-propanediol’s strong self-affinity (−14.72 kJ·mol^–1^) and the overall DES composition. The *x*_*AA*_ profile reaches around 0.5, similar to AChCl:1,2-PG, due to the identical AA affinity (−15.21 kJ·mol^–1^). The *x*_*AB*_ profile peaks at approximately 0.5, the highest among all systems, consistent with the strongest AB affinity (−15.06 kJ·mol^–1^), indicating optimal compatibility between acetylcholine chloride and 1,3-propanediol. The *x*_*BW*_ and *x*_*AW*_ profiles also show slight increases compared to AChCl:1,2-PG, reaching around 0.2 and 0.15, respectively, reflecting the enhanced BW (−11.63 kJ·mol^–1^) and AW (−11.31 kJ·mol^–1^) affinities.

Hence, the concentration profiles highlight distinct molecular organization patterns across the four DESs. Both choline chloride-based DES show a dominance of BB interactions, with ChCl:1,3-PG exhibiting stronger BB and AB associations due to 1,3-propanediol’s favorable properties. The acetylcholine chloride-based DES display enhanced AA and AB interactions, with AChCl:1,3-PG showing the most balanced and strongest heteromolecular associations due to the synergistic combination of acetylcholine chloride and 1,3-propanediol. Water interactions (AW, BW) remain relatively weak across all systems, but acetylcholine chloride-based DESs exhibit slightly higher solvation tendencies, particularly with water-salt (AW) contacts. These differences underscore the role of molecular structure in dictating the interaction hierarchy and distribution of DES constituents in aqueous environments.

### 4.7 Density Modeling

It is interesting to investigate whether intermolecular interactions that significantly affect concentration profiles can serve as predictors of the measured physicochemical properties. To explore this, an effort was made to identify linear relationships between measured density and parameters that quantify these intermolecular interactions. The concentration-dependent Gibbs free energies (*G*_*r(x)*_) were employed as potential descriptors, alongside their decomposition into enthalpic and entropic components, expressed as Δ*G*_*r*(*x*)_ = Δ*H*_*r*(*x*)_ – *T* · Δ*S*_*r*(*x*)_. This data set, which encompasses the characteristics of all possible pair formations in the studied DESs, was further enriched by incorporating computed values of the σ-potential distributions. These values, directly obtainable through COSMO-RS computations, capture temperature-related trends and have previously been utilized in various machine learning protocols. The σ-potential is particularly effective in reflecting the diverse contributions of charge density distributions. It should be noted that the σ–potential values for each monomer or pair were averaged based on the solution composition. Consequently, the distributions shown in Figure 5 also reflect the weights used to calculate the composition-dependent σ-potential, expressed as, μ_*i*_ = ∑*x*_*i*_ · μ_*i*_ in a simplified form represented by 12 points.

To identify the most effective combination of molecular descriptors, a Python script was developed using the “LinearRegression” library from the “sklearn” scientific package.^56^ The total number of descriptors considered was substantial (60) yet manageable enough to allow an exhaustive exploration of all possible combinations, ranging from one to six descriptors. It was found that exceeding this threshold was unnecessary, as evidenced by the results of this comprehensive search presented in Figure 6. The figure demonstrates that increasing the number of descriptors in the linear model consistently improves the overall accuracy, as indicated by the adjusted squared correlation coefficient. However, no significant improvement is achieved by including more than five descriptors, as a saturation point is observed. Thus, a five-descriptor model strikes a reasonable balance between the complexity of the linear equation and the model’s accuracy. The final formula corresponding to best best-performing model is the following (eq 13):

13 $$d={a_{1}\cdot \Delta G}_{r\left(x\right)}^{\mathrm{BB}}+a_{2}\cdot {\Delta H}_{r\left(x\right)}^{\mathrm{BB}}+a_{3}\cdot {\Delta H}_{r\left(x\right)}^{\mathrm{BW}}+a_{4}\cdot {\Delta S}_{r\left(x\right)}^{\mathrm{AA}}+a_{5}\cdot {H\mathrm{BD}}_{1}+a_{0}$$

­(*a*_0_ = 536.4513074600069, *a*_1_ = –27.65851974637204, *a*_2_ = –17.162062949599903, *a*_3_ = 19.278446881823978, *a*_4_ = 0.5595847558332535, *a*_5_ = –225.60907332138297)

**Figure 6:**
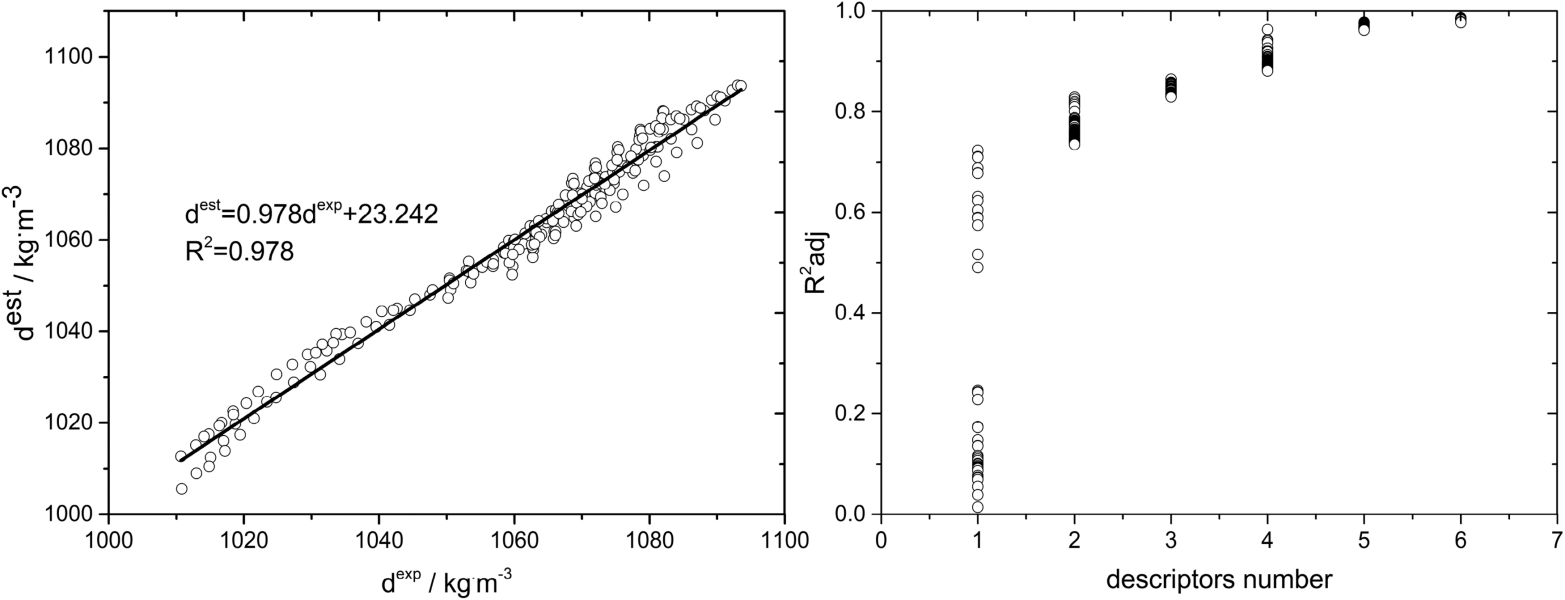
Characteristics of predictive potential of generated models resulting from all combinations of molecular descriptors. Only statistically significant models (*p* < 0.05) with the highest *R*_adj_^2^ were included.

N = 262, *p* < 0.001, *R*_adj_^2^ = 0.977 (adjusted), *R*_CV_^2^ = 0,966 (5-fold cross validation)

The best model found among all screened out relies on contributions from affinity (Δ*G*_*r*(*x*)_^*BB*^) and energetics (Δ*H*_*r*(*x*)_^*BB*^) of HBD interactions, enthalpy (Δ*H*_*r*(*x*)_^*BW*^) of HBD-W attractions, entropic (Δ*H*_*r*(*x*)_^*BW*^) contribution of HBA dimerization and doniticity of the region of σ-potential between −0.3 and −0.25 e·Å^–2^ σ values. To the author’s knowledge, a comprehensive analysis of DES density in terms of direct contact has not been reported in the scientific literature so far. The model is robust, possesses predictive power, and might be applied to other systems within the applicability domain, as documented in Figure 7.

**Figure 7:**
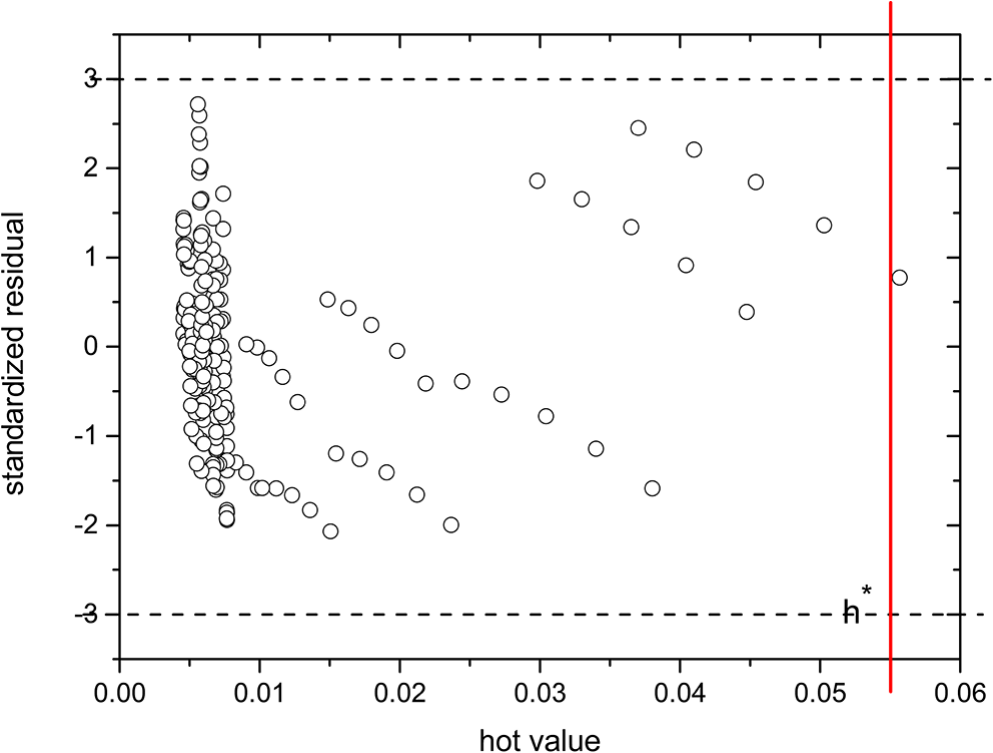
Applicability domain represented by plotting the standardized residuals as a function of the hat values, where h* stands the threshold value characterizing the model defined with an eq 13.

The selected five-descriptor model is not merely a statistical construct but reflects the underlying physics governing density in these complex mixtures. The selection of descriptors related primarily to the hydrogen bond donor (HBD, the glycol) highlights its dominant role in determining liquid structure and packing. The values of Δ*G*_*r*(*x*)_^*BB*^ and Δ*H*_*r*(*x*)_^*BW*^ quantify the strength and nature of the glycol–glycol interactions. A more negative self-association promotes a more extensive, internally bonded network within the DES, leading to a specific, often less compact, packing efficiency that directly influences density. Enthalpy of glycol-water interactions, Δ*H*_*r*(*x*)_^*BW*^, capture the energetic favorability of the glycol hydrating versus self-associating. A more exothermic value indicates a strong driving force for water to integrate into and disrupt the glycol network, leading to a new, mixed packing structure with a characteristic density. Entropy of HBA self-association, Δ*S*_*r*(*x*)_^*AA*^, reflects the change in disorder associated with this process. This descriptor likely acts as a proxy for the conformational flexibility of the HBA cation (ChCl vs AChCl), which affects how efficiently the ions and glycol molecules can pack together. Finally, hydrogen bond donor strength, *HBD*_1_, in a lowest σ-potential region directly quantifies the HBD’s ability to donate a hydrogen bond. A stronger HBD character (more negative value) will lead to stronger and potentially more numerous intermolecular bonds, which constrains the molecular packing and thus impacts the density.

The selected descriptors in eq 13 offer physical insights into the density of aqueous DESs. The negative contribution from of Δ*G*_*r*(*x*)_^*BB*^ (affinity of HBD interactions) implies that stronger HBD self-association (more negative values) leads to lower density by encouraging segregated domains and less compact packing, as seen in 1,3-PG systems with their enhanced BB affinities (−14.72 kJ·mol^–1^). The energetics term for HBD interactions (negative coefficient) highlights van der Waals and electrostatic contributions that densify the mixture through favorable homomolecular alignment. The enthalpic component of HBD-W attractions (negative) underscores exothermic solvation that tightens the structure, increasing density, while the entropic contribution to HBA dimerization (positive) indicates that entropy loss in HBA clusters (e.g., due to steric constraints in AChCl) enhances packing efficiency. Finally, the donicity in the σ-potential region (−0.3 to −0.25 e·Å^–2^, negative coefficient) reflects stronger HBA capabilities, fostering denser hydrogen-bond networks.

## 5 Conclusions

In this work, an integrated multiscale study combining high-precision experiments, thermodynamic theory, and computational quantum chemistry was conducted to elucidate the behavior of aqueous solutions of four DESs based on choline or acetylcholine chlorides with 1,2- and 1,3-propanediols. The experimental determination of density and speed of sound allowed for the calculation of key excess thermodynamic properties (*V*_*m*_^*E*^, *K*_*S*,*m*_^*E*^). These were consistently negative across all compositions and temperatures, providing macroscopic evidence of strong, specific interactions—predominantly hydrogen bonding—between DES and water molecules. The strength of these interactions was found to be highly sensitive to molecular structure, following the order: AChCl:1,2-PG > ChCl:1,2-PG > AChCl:1,3-PG > ChCl:1,3-PG. This trend demonstrates that both the nature of the HBA (with the acetyl group in AChCl enhancing interactions) and the isomeric form of the glycol (with the 1,2-isomer facilitating better mixing with water) are critical determinants of mixture properties. The application of the Prigogine-Flory-Patterson (PFP) theory provided a rigorous theoretical framework for interpreting the volumetric data. The analysis confirmed that the negative excess molar volumes are dominated by the interactional contribution, with free volume and internal pressure effects playing a minor role. The negative values of the interaction parameter, χ_12_, and their systematic variation with DES composition, quantitatively support the conclusion that DES-water interactions are more favorable than the self-associations within the pure components. These macroscopic and theoretical findings were rationalized at the molecular level using COSMO-RS. The analysis of pairwise affinities revealed that the 1,3-propanediol isomer and acetylcholine chloride have a greater propensity for self-association (stronger BB and AA affinities), which explains their weaker net interaction with water observed experimentally. The development of a speciation model based on concentration-dependent equilibrium constants provided unprecedented insight into the shifting molecular landscape as a function of composition. The calculated concentration profiles of homo- and heteromolecular pairs directly illustrated how the balance between self-association (AA, BB) and cross-association (AB) differs between DESs, governing their distinct solvation environments in water.

Finally, this deep molecular understanding was leveraged for predictive purposes. A robust linear regression model for estimating density was developed using descriptors derived directly from the COSMO-RS computations. The model’s high accuracy and the physical significance of its selected descriptors—which highlight the dominant role of glycol self-association and hydration—demonstrate a successful pathway toward the in silico design and screening of DES mixtures with tailored properties. This study successfully bridges scales from macroscopic properties to molecular interactions, offering a consistent and predictive framework for understanding the structure–property relationships in aqueous deep eutectic solvents. The methodologies applied, particularly the speciation model and the descriptor-based density prediction, provide powerful tools for the future rational design of these versatile solvents. Furthermore, the COSMO-RS-based linear regression model for density prediction holds practical implications for DES design, enabling rapid solvent screening, formulation optimization in pharmaceutical and extraction applications, and reduced reliance on empirical testing.

## Supplementary Material



## Notes

Values of the excess molar volume, excess molar compressibility, parameters of the Redlich – Kister equations with the corresponding root-mean-square deviations for *V*_*m*_^*E*^ and for *K*_*S,m*_^*E*^ for the studied aqueous mixtures, values of the partial molar volumes of DESs and water in the studied binary mixtures at *T* = (293.15 to 313.15) K and atmospheric pressure (0.1 MPa), as well as the isobaric thermal expansion coefficients, isochoric molar heat capacities, and Prigogine-Flory-Patterson theory parameters of DESs and water, together with equations defining them (PDF)
