# Supplementary material for: Dissecting Molecular Interactions in Aqueous Deep Eutectic Solvents: A Multi-Scale Study of Choline- and Acetylcholine-Based Propylene Glycol Isomers
Source: J Phys Chem B. 2026 Feb 16;130(8):2402–19. doi: 10.1021/acs.jpcb.5c08377 (PMC13298888; doi:10.1021/acs.jpcb.5c08377)
Supplement: Supplementary file 1 [file jp5c08377_si_001.pdf]

Supporting Information

Dissecting Molecular Interactions in Aqueous Deep Eutectic Solvents: A Multi-Scale Study of Choline- and Acetylcholine-Based Propylene Glycol Isomers

Dorota Warmińska[a], Adrianna Sutkowska[a], Marzena Jamrógiewicz[b], Piotr Cysewski[c]

[a] Department of Physical Chemistry, Faculty of Chemistry, Gdańsk University of Technology, ul. Narutowicza 11/12, 80-233 Gdańsk, Poland

[b] Department of Physical Chemistry, Faculty of Pharmacy, Medical University of Gdańsk, Al. Gen. Hallera 107, 80-416 Gdańsk, POLAND

[c] Department of Physical Chemistry, Pharmacy Faculty, Collegium Medicum of Bydgoszcz, Nicolaus Copernicus University in Toruń, Kurpińskiego 5, 85-096 Bydgoszcz, Poland

*Corresponding author: e-mail address: dorwarmi@pg.edu.pl

Table S1. Excess molar volumes, V_m^E, and excess molar compressibilities, $K_{S,m}^E$, of ChCl:1,2-PG and water in their binary mixtures at T = (293.15 to 313.15) K and atmospheric pressure (0.1 MPa).

T / K	293.15 K	298.15 K	303.15 K	308.15 K	313.15 K
x_1	$10^6 V_m^E$ / (m^3·mol^{-1})				
0.0000	0.000	0.00	0.00	0.00	0.00
0.0494	-0.12	-0.12	-0.12	-0.11	-0.11
0.0984	-0.27	-0.26	-0.25	-0.24	-0.23
0.1903	-0.48	-0.46	-0.44	-0.42	-0.41
0.2855	-0.58	-0.56	-0.54	-0.53	-0.51
0.3790	-0.61	-0.59	-0.57	-0.56	-0.54
0.5061	-0.57	-0.56	-0.54	-0.53	-0.51
0.5982	-0.49	-0.48	-0.47	-0.46	-0.44
0.7007	-0.40	-0.39	-0.38	-0.38	-0.37
0.7886	-0.29	-0.28	-0.28	-0.27	-0.27
0.9072	-0.13	-0.13	-0.13	-0.12	-0.12
0.9453	-0.08	-0.08	-0.07	-0.07	-0.08
1.0000	0.00	0.00	0.00	0.00	0.00
x_1	$10^{14} K_{S,m}^E$/ (m^5·N^{-1}·mol^{-1})				
0.0000	0.00	0.00	0.00	0.00	0.00
0.0494	-0.14	-0.13	-0.12	-0.11	-0.11
0.0984	-0.23	-0.21	-0.20	-0.19	-0.18
0.1903	-0.32	-0.30	-0.29	-0.28	-0.26
0.2855	-0.34	-0.33	-0.32	-0.30	-0.29
0.3790	-0.34	-0.32	-0.31	-0.30	-0.29
0.5061	-0.30	-0.29	-0.28	-0.27	-0.26
0.5982	-0.26	-0.25	-0.24	-0.23	-0.23
0.7007	-0.20	-0.19	-0.18	-0.18	-0.18
0.7886	-0.13	-0.12	-0.12	-0.12	-0.11
0.9072	-0.06	-0.06	-0.06	-0.06	-0.06
0.9453	-0.04	-0.04	-0.04	-0.04	-0.04
1.0000	0.00	0.00	0.00	0.00	0.00

[a] Standard uncertainties u are $u(T)$ = 0.01 K, $u(p)$ = 10 kPa, and the combined standard uncertainty $u(x_1)$ = 0.0012, $u(V_m^E)$ = 0.011·10^{-6} m^3·mol^{-1}, $u(K_{S,m}^E)$ = 0.018·10^{-14} m^5·N^{-1}·mol^{-1}

Table S2. Excess molar volumes, V_m^E, and excess molar compressibilities, $K_{S,m}^E$, of ChCl:1,3-PG and water in their binary mixtures at T = (293.15 to 313.15) K and atmospheric pressure (0.1 MPa).

T / K	293.15 K	298.15 K	303.15 K	308.15 K	313.15 K
x_1	$10^6 V_m^E$ / ($m^3 \cdot mol^{-1}$)				
0.0000	0.00	0.00	0.00	0.00	0.00
0.0509	-0.07	-0.07	-0.06	-0.06	-0.06
0.1028	-0.15	-0.14	-0.14	-0.13	-0.13
0.2002	-0.29	-0.27	-0.26	-0.25	-0.24
0.2953	-0.37	-0.36	-0.35	-0.34	-0.33
0.4056	-0.40	-0.38	-0.37	-0.36	-0.35
0.4882	-0.39	-0.38	-0.37	-0.36	-0.36
0.5988	-0.35	-0.34	-0.33	-0.33	-0.32
0.6853	-0.29	-0.28	-0.28	-0.27	-0.27
0.7792	-0.22	-0.21	-0.21	-0.20	-0.20
0.8793	-0.12	-0.12	-0.12	-0.12	-0.11
0.9377	-0.06	-0.05	-0.05	-0.05	-0.05
1.0000	0.00	0.00	0.00	0.00	0.00
x_1	$10^{14} K_{S,m}^E$/ ($m^5 \cdot N^{-1} \cdot mol^{-1}$)				
0.0000	0.00	0.00	0.00	0.00	0.00
0.0509	-0.10	-0.09	-0.09	-0.08	-0.08
0.1028	-0.18	-0.16	-0.15	-0.14	-0.13
0.2002	-0.25	-0.23	-0.22	-0.21	-0.20
0.2953	-0.27	-0.26	-0.24	-0.23	-0.22
0.4056	-0.26	-0.25	-0.24	-0.23	-0.22
0.4882	-0.24	-0.23	-0.22	-0.21	-0.21
0.5988	-0.20	-0.19	-0.18	-0.18	-0.18
0.6853	-0.17	-0.17	-0.16	-0.15	-0.16
0.7792	-0.12	-0.12	-0.11	-0.11	-0.11
0.8793	-0.06	-0.06	-0.06	-0.06	-0.06
0.9377	-0.04	-0.03	-0.03	-0.03	-0.04
1.0000	0.00	0.00	0.00	0.00	0.00

[a] Standard uncertainties u are $u(T)$ = 0.01 K, $u(p)$ = 10 kPa, and the combined standard uncertainty $u(x_1)$ = 0.0012, $u(V_m^E)$) = $0.019 \cdot 10^{-6}$ $m^3 \cdot mol^{-1}$, $u(K_{S,m}^E)$ = $0.018 \cdot 10^{-14}$ $m^5 \cdot N^{-1} \cdot mol^{-1}$

Table S3. Excess molar volumes, V_m^E, and excess molar compressibilities, $K_{S,m}^E$, of AChCl:1,2-PG and water in their binary mixtures at T = (293.15 to 313.15) K and atmospheric pressure (0.1 MPa).

T / K	293.15 K	298.15 K	303.15 K	308.15 K	313.15 K
x_1	$10^6 V_m^E$ / (m^3·mol^{-1})				
0.0000	0.00	0.00	0.00	0.00	0.00
0.0498	-0.15	-0.15	-0.14	-0.14	-0.13
0.0991	-0.33	-0.31	-0.30	-0.29	-0.28
0.1993	-0.55	-0.55	-0.51	-0.49	-0.47
0.2951	-0.65	-0.62	-0.60	-0.58	-0.56
0.3918	-0.66	-0.64	-0.62	-0.59	-0.57
0.4946	-0.61	-0.60	-0.58	-0.57	-0.55
0.5931	-0.54	-0.52	-0.50	-0.48	-0.47
0.6904	-0.43	-0.42	-0.41	-0.40	-0.39
0.7902	-0.30	-0.29	-0.29	-0.28	-0.28
0.8787	-0.18	-0.18	-0.18	-0.18	-0.17
0.9640	-0.05	-0.04	-0.05	-0.05	-0.05
1.0000	0.00	0.00	0.00	0.00	0.00
x_1	$10^{14} K_{S,m}^E$ / (m^5·N^{-1}·mol^{-1})				
0.0000	0.00	0.00	0.00	0.00	0.00
0.0498	-0.16	-0.15	-0.14	-0.13	-0.12
0.0991	-0.26	-0.24	-0.23	-0.22	-0.21
0.1993	-0.36	-0.34	-0.32	-0.31	-0.30
0.2951	-0.38	-0.37	-0.35	-0.34	-0.33
0.3918	-0.36	-0.34	-0.33	-0.32	-0.31
0.4946	-0.32	-0.31	-0.30	-0.29	-0.28
0.5931	-0.28	-0.27	-0.26	-0.25	-0.24
0.6904	-0.21	-0.20	-0.19	-0.19	-0.18
0.7902	-0.14	-0.14	-0.13	-0.13	-0.12
0.8787	-0.08	-0.07	-0.07	-0.07	-0.07
0.9640	-0.02	-0.02	-0.01	-0.01	-0.01
1.0000	0.00	0.00	0.00	0.00	0.00

[a] Standard uncertainties u are $u(T)$ = 0.01 K, $u(p)$ = 10 kPa, and the combined standard uncertainty $u(x_1)$ = 0.0012, $u(V_m^E)$ = 0.019·10^{-6} m^3·mol^{-1}, $u(K_{S,m}^E)$ = 0.018·10^{-15} m^5·N^{-1}·mol^{-1}

Table S4. Excess molar volumes, V_m^E, and excess molar compressibilities, $K_{S,m}^E$, of AChCl:1,3-PG and water in their binary mixtures at T = (293.15 to 313.15) K and atmospheric pressure (0.1 MPa).

T / K	293.15 K	298.15 K	303.15 K	308.15 K	313.15 K
x_1	$10^6 V_m^E$ / (m^3·mol^{-1})				
0.0000	0.00	0.00	0.00	0.00	0.00
0.0498	-0.08	-0.07	-0.07	-0.06	-0.06
0.0997	-0.18	-0.17	-0.16	-0.15	-0.14
0.1955	-0.34	-0.32	-0.31	-0.29	-0.28
0.2923	-0.44	-0.42	-0.40	-0.39	-0.37
0.3902	-0.47	-0.45	-0.43	-0.41	-0.39
0.5041	-0.44	-0.43	-0.41	-0.40	-0.38
0.5958	-0.41	-0.37	-0.36	-0.35	-0.35
0.6948	-0.31	-0.31	-0.30	-0.29	-0.28
0.7852	-0.23	-0.23	-0.22	-0.21	-0.21
0.8801	-0.13	-0.13	-0.13	-0.12	-0.12
0.9661	-0.04	-0.04	-0.04	-0.04	-0.03
1.0000	0.00	0.00	0.00	0.00	0.00
x_1	$10^{14} K_{S,m}^E$ / (m^5·N^{-1}·mol^{-1})				
0.0000	0.00	0.00	0.00	0.00	0.00
0.0498	-0.12	-0.11	-0.10	-0.09	-0.09
0.0997	-0.20	-0.18	-0.17	-0.16	-0.15
0.1955	-0.28	-0.26	-0.24	-0.23	-0.22
0.2923	-0.30	-0.29	-0.27	-0.26	-0.24
0.3902	-0.29	-0.28	-0.27	-0.25	-0.24
0.5041	-0.26	-0.25	-0.24	-0.23	-0.22
0.5958	-0.23	-0.22	-0.21	-0.20	-0.19
0.6948	-0.18	-0.17	-0.16	-0.15	-0.15
0.7852	-0.13	-0.12	-0.12	-0.11	-0.11
0.8801	-0.07	-0.07	-0.07	-0.06	-0.06
0.9661	-0.02	-0.02	-0.02	-0.02	-0.02
1.0000	0.00	0.00	0.00	0.00	0.00

[a] Standard uncertainties u are $u(T)$ = 0.01 K, $u(p)$ = 10 kPa, and the combined standard uncertainty $u(x_1)$ = 0.0012, $u(V_m^E)$ = 0.019·10^{-6} m^3·mol^{-1}, $u(K_{S,m}^E)$ = 0.018·10^{-15} m^5·N^{-1}·mol^{-1}

Table S5 - Parameters A_i of the Redlich - Kister equation and the corresponding root mean square deviations, RMSD, for V_m^E/(m^3·mol^{-1}) for the binary mixtures at T = (293.15 to 313.15) K and atmospheric pressure (0.1 MPa)[a].

T /K	$10^6 A_0$	$10^6 A_1$	$10^6 A_2$	$10^6 A_3$	10^6 RMSD
	ChCl:1,2-PG (1) +water (2)				
293.15	-2.322	1.291	-0.039	-0.620	0.011
298.15	-2.259	1.213	-0.018	-0.586	0.009
303.15	-2.197	1.140	-0.006	-0.551	0.009
308.15	-2.137	1.074	-0.003	-0.518	0.009
313.15	-2.078	1.012	-0.004	-0.487	0.009
	ChCl:1,3-PG (1) +water(2)				
293.15	-1.589	0.613	0.355	-0.431	0.010
298.15	-1.544	0.561	0.366	-0.408	0.009
303.15	-1.504	0.513	0.370	-0.385	0.009
308.15	-1.467	0.470	0.372	-0.364	0.009
313.15	-1.432	0.432	0.371	-0.346	0.008
	AChCl:1,2-PG (1) + water(2)				
293.15	-2.449	1.521	-0.380	-0.516	0.012
298.15	-2.377	1.467	-0.403	-0.505	0.015
303.15	-2.304	1.361	-0.336	-0.527	0.011
308.15	-2.230	1.292	-0.330	-0.536	0.012
313.15	-2.154	1.225	-0.320	-0.521	0.012
	AChCl:1,3-PG (1) + water(2)				
293.15	-1.760	1.017	0.091	-0.960	0.012
298.15	-1.694	0.946	0.111	-0.939	0.013
303.15	-1.635	0.907	0.126	-0.953	0.013
308.15	-1.574	0.843	0.141	-0.914	0.012
313.15	-1.525	0.728	0.229	-0.670	0.017

Table S6. Parameters A_i of the Redlich - Kister equation and the corresponding root mean square deviations, RMSD, for $K_{S,m}^E$ /($m^5 \cdot mol^{-1} \cdot N^{-1}$) for the binary mixtures at T = (293.15 to 313.15) K and atmospheric pressure (0.1 MPa)[a]

T /K	$10^{14}A_0$	$10^{14}A_1$	$10^{14}A_2$	$10^{14}A_3$	10^{14} RMSD
	ChCl:1,2-PG (1) +water (2)				
293.15	-1.1685	0.8560	-0.7836	0.4181	0.0065
298.15	-1.1293	0.8213	-0.6951	0.3417	0.0060
303.15	-1.0957	0.7901	-0.6156	0.2800	0.0057
308.15	-1.0640	0.7509	-0.5565	0.2404	0.0054
313.15	-1.0338	0.7163	-0.5084	0.2108	0.0054
	ChCl:1,3-PG (1) +water(2)				
293.15	-0.9487	0.6472	-0.5319	0.2137	0.0043
298.15	-0.9203	0.5514	-0.3712	0.3225	0.0022
303.15	-0.8787	0.5312	-0.3379	0.228	0.0024
308.15	-0.8434	0.4937	-0.3893	0.2059	0.0049
313.15	-0.8253	0.4538	-0.3168	0.1119	0.0027
	AChCl:1,2-PG (1) + water(2)				
293.15	-1.2652	0.8787	-0.8114	0.8309	0.0034
298.15	-1.2247	0.858	-0.7141	0.7122	0.0030
303.15	-1.1923	0.8017	-0.5889	0.7078	0.0039
308.15	-1.1601	0.7776	-0.5164	0.64	0.0039
313.15	-1.1319	0.7459	-0.4536	0.5975	0.0044
	AChCl:1,3-PG (1) + water(2)				
293.15	-1.0436	0.6835	-0.6181	0.4403	0.0037
298.15	-0.9963	0.6459	-0.5384	0.3616	0.0026
303.15	-0.9558	0.6011	-0.4685	0.313	0.0020
308.15	-0.9125	0.5736	-0.4131	0.2597	0.0016
313.15	-0.8759	0.5419	-0.3735	0.2158	0.0017

Table S7. Partial molar volumes, $\overline{V}_i$, of DESs and water in their binary mixtures at T = (293.15 to 313.15) K and atmospheric pressure (0.1 MPa).

	$10^6\overline{V}_1$ / ($m^3 \cdot mol^{-1}$)	$10^6\overline{V}_2$ / ($m^3 \cdot mol^{-1}$)[1]	$10^6\overline{V}_1$ / ($m^3 \cdot mol^{-1}$)	$10^6\overline{V}_2$ / ($m^3 \cdot mol^{-1}$)	$10^6\overline{V}_1$ / ($m^3 \cdot mol^{-1}$)	$10^6\overline{V}_2$ / ($m^3 \cdot mol^{-1}$)	$10^6\overline{V}_1$ / ($m^3 \cdot mol^{-1}$)	$10^6\overline{V}_2$ / ($m^3 \cdot mol^{-1}$)	$10^6\overline{V}_1$ / ($m^3 \cdot mol^{-1}$)	$10^6\overline{V}_2$ / ($m^3 \cdot mol^{-1}$)
T /K	293.15 K		298.15 K		303.15 K		308.15 K		313.15 K	
x_1	ChCl:1,2-PG (1) +water (2)									
0.0000	82.78	18.05	83.15	18.07	83.51	18.09	83.86	18.12	84.19	18.16
0.0494	83.03	18.04	83.38	18.06	83.73	18.09	84.07	18.12	84.40	18.15
0.0984	83.34	18.02	83.68	18.04	84.01	18.06	84.34	18.10	84.67	18.13
0.1903	84.00	17.90	84.31	17.93	84.61	17.96	84.91	18.00	85.22	18.04
0.2855	84.65	17.70	84.92	17.74	85.20	17.78	85.48	17.82	85.76	17.87
0.3790	85.15	17.46	85.40	17.50	85.66	17.55	85.92	17.60	86.18	17.66
0.5061	85.56	17.13	85.81	17.19	86.05	17.25	86.30	17.31	86.55	17.37
0.5982	85.71	16.96	85.95	17.02	86.19	17.08	86.44	17.14	86.68	17.21
0.7007	85.76	16.86	86.01	16.91	86.25	16.97	86.50	17.03	86.75	17.09
0.7886	85.78	16.82	86.02	16.87	86.27	16.92	86.52	16.97	86.77	17.03
0.9072	85.79	16.71	86.04	16.75	86.29	16.80	86.54	16.85	86.79	16.90
0.9453	85.80	16.61	86.05	16.65	86.30	16.70	86.55	16.76	86.80	16.81
1.0000	85.81	16.36	86.05	16.42	86.30	16.48	86.55	16.54	86.80	16.60
ChCl:1,3-PG (1) +water (2)										
0.0000	83.63	18.05	83.94	18.07	84.23	18.09	84.51	18.12	84.79	18.16
0.0509	83.56	18.05	83.86	18.07	84.14	18.10	84.42	18.13	84.69	18.16

0.1028	83.61	18.05	83.89	18.07	84.16	18.09	84.43	18.12	84.70	18.16
0.2002	83.87	18.00	84.13	18.02	84.38	18.05	84.64	18.09	84.89	18.12
0.2953	84.21	17.88	84.45	17.91	84.69	17.95	84.93	17.99	85.16	18.03
0.4056	84.58	17.69	84.80	17.73	85.03	17.77	85.25	17.81	85.48	17.86
0.4882	84.78	17.52	85.00	17.57	85.22	17.61	85.44	17.66	85.66	17.71
0.5988	84.95	17.32	85.17	17.37	85.39	17.42	85.60	17.47	85.82	17.52
0.6853	85.01	17.21	85.23	17.26	85.45	17.31	85.67	17.36	85.89	17.41
0.7792	85.04	17.15	85.26	17.19	85.48	17.23	85.70	17.28	85.92	17.33
0.8793	85.04	17.12	85.27	17.15	85.49	17.19	85.71	17.23	85.93	17.28
0.9377	85.05	17.08	85.27	17.12	85.49	17.16	85.71	17.20	85.93	17.24
1.0000	85.05	17.00	85.27	17.04	85.49	17.09	85.71	17.13	85.93	17.18
AChCl:1,2-PG (1) +water (2)										
0.0000	90.78	18.05	91.17	18.07	91.73	18.09	92.18	18.12	92.61	18.16
0.0498	91.31	18.03	91.69	18.06	92.18	18.08	92.60	18.11	93.01	18.15
0.0991	91.84	17.99	91.21	18.01	92.63	18.05	93.02	18.08	93.41	18.11
0.1993	92.80	17.82	93.15	17.85	93.50	17.89	93.85	17.93	94.20	17.98
0.2951	93.54	17.58	93.86	17.62	94.17	17.67	94.49	17.72	94.82	17.77
0.3918	94.05	17.32	94.36	17.36	94.65	17.43	94.96	17.48	95.27	17.54
0.4946	94.37	17.07	94.67	17.12	94.96	17.19	95.25	17.25	95.60	17.32
0.5931	94.51	16.90	94.81	16.96	95.09	17.03	95.38	17.10	95.68	17.17
0.6904	94.56	16.82	94.85	16.88	95.14	16.95	95.43	17.02	95.72	17.09
0.7902	94.58	16.78	94.87	16.84	95.16	16.90	95.45	16.97	95.74	17.04
0.8787	94.59	16.69	94.88	16.75	95.18	16.80	95.47	16.86	95.76	16.92

0.9640	94.61	16.43	94.90	16.47	95.20	16.51	95.49	16.55	95.79	16.62
1.0000	94.62	16.22	94.91	16.25	95.20	16.29	95.50	16.32	95.79	16.39
AChCl:1,3-PG (1) +water (2)										
0.0000	91.82	18.05	92.21	18.07	92.60	18.09	92.96	18.12	93.23	18.16
0.0498	91.69	18.05	92.07	18.07	92.42	18.10	92.78	18.13	93.11	18.16
0.0997	91.75	18.05	92.10	18.07	92.43	18.10	92.77	18.13	93.12	18.16
0.1955	92.13	17.98	92.44	18.01	92.74	18.04	93.05	18.07	93.39	18.11
0.2923	92.62	17.81	92.91	17.85	93.19	17.89	93.48	17.94	93.77	17.98
0.3902	93.06	17.59	93.32	17.64	93.59	17.68	93.86	17.74	94.12	17.80
0.5041	93.37	17.35	93.62	17.40	93.89	17.45	94.15	17.51	94.39	17.59
0.5958	93.47	17.22	93.73	17.28	93.99	17.33	94.25	17.39	94.50	17.45
0.6948	93.50	17.18	93.76	17.23	94.01	17.29	94.27	17.35	94.55	17.38
0.7852	93.50	17.18	93.76	17.23	94.01	17.29	94.28	17.34	94.56	17.35
0.8801	93.51	17.09	93.77	17.14	94.03	17.20	94.29	17.25	94.57	17.29
0.9661	93.54	16.72	93.80	16.77	94.06	16.82	94.32	16.89	94.58	17.08
1.0000	93.54	16.44	93.80	16.49	94.06	16.54	94.32	16.62	94.59	16.92

Equations Defining the Prigogine-Flory-Patterson Theory Parameters

The reduced volume for pure substance i is defined in terms of the thermal expansion coefficients, α_i, as:

$$\tilde{V}_i = \left(\frac{1+\frac{4}{3}\alpha_i T}{1+\alpha_i T}\right)^3 \quad \text{(S1)}$$

The reduced volume of the mixture, $\tilde{V}$, is calculated from:

$$\tilde{V} = \psi_1\widetilde{V_1} + \psi_2\widetilde{V_2} \quad \text{(S2)}$$

where the molecular contact energy fraction, ψ, is expressed by: $\psi_1 = 1-\psi_2 = \frac{\phi_1 p_1^*}{\phi_1 p_1^* + \phi_2 p_2^*}$ with the hardcore volume fraction, Φ, calculated from $\phi_1 = 1-\phi_2 = \frac{x_1 V_1^*}{x_1 V_1^* + x_2 V_2^*}$.

The characteristic volume, V_i^*, is calculated from the molar volume from the expression

$V_i^* = \frac{V_i^0}{\widetilde{V}_i}$ and the characteristic pressure is expressed by:

$$p_i^* = \frac{\alpha_i}{\kappa_{Ti}} T\widetilde{V}_i^2 \quad \text{(S3)}$$

where κ_{Ti} is the isothermal compressibility obtained from the isentropic compressibility from the thermodynamic relation:

$$\kappa_{Ti} = \kappa_{Si} + \frac{V_i^0 \alpha_i^2 T}{C_{pi}} \quad \text{(S4)}$$

with the isobaric heat capacity C_{pi}.

The molecular surface fraction of component 2 is given by: $\Theta_2 = \frac{\phi_2}{\phi_1 \frac{s_1}{s_2} + \phi_2}$, in which the ratio of the surface contact sites per segment is given by:

$$\frac{s_1}{s_2} = \left(\frac{v_2^*}{v_1^*}\right)^{1/3} \quad \text{(S5)}$$

Table S8 Isobaric thermal expansion coefficient (α_p), isochoric molar heat capacity(C_P), Prigogine-Flory-Patterson theory parameters: characteristic volume(V*), reduce volume ($\tilde{V}$), characteristic pressure (P*), and ratio of molecular surface to volume ratio (S_1/S_2) of DES to water.

DES	T / K	$10^4 \alpha$ / K^{-1}	C_p / ($J \cdot mol^{-1} \cdot K^{-1}$)	$10^6 V^*$ / ($m^{-3} \cdot mol^{-1}$)	$\tilde{V}$	$10^8 P^*$ / Pa	S_1/S_2
ChCl:1,2-PG							
	293.15	5.74	198.2	74.547	1.151	6.340	0.611
	298.15	5.75	199.6	74.597	1.154	6.379	0.609
	303.15	5.76	201.0	74.648	1.156	6.415	0.607
	308.15	5.77	203.3	74.699	1.159	6.651	0.604
	313.15	5.78	205.6	74.753	1.161	6.690	0.602
ChCl:1,3-PG							
	293.15	5.18	195.4	74.753	1.138	6.290	0.611
	298.15	5.18	197.7	74.816	1.140	6.328	0.608
	303.15	5.17	200.0	74.880	1.142	6.363	0.606

	308.15	5.16	202.3	74.945	1.142	6.397	0.604
	313.15	5.16	204.6	75.011	1.146	6.408	0.601
AChCl:1,2-PG							
	293.15	6.20	218.0	81.442	1.162	6.624	0.594
	298.15	6.18	220.1	81.544	1.164	6.633	0.591
	303.15	6.17	221.6	81.648	1.166	6.637	0.589
	308.15	6.16	223.2	81.754	1.166	6.637	0.586
	313.15	6.14	225.7	81.859	1.170	6.637	0.584
AChCl:1,3-PG							
	293.15	5.55	218.5	81.592	1.146	6.568	0.593
	298.15	5.54	221.1	81.671	1.149	6.599	0.591
	303.15	5.53	223.7	81.751	1.151	6.627	0.588
	308.15	5.53	226.2	81.832	1.151	6.656	0.586
	313.15	5.52	229.8	81.914	1.155	6.683	0.584
Water							
	293.15	2.120	75.4	17.031	1.060	1.520	-
	298.15	2.565	75.3	16.843	1.073	1.945	-
	303.15	3.010	75.3	16.661	1.086	2.405	-
	308.15	3.455	75.2	16.485	1.099	2.898	-
	313.15	3.901	75.2	16.315	1.113	3.420	-
